# Predicting the topography of fitness landscapes from the structure of genotype–phenotype maps

**DOI:** 10.1093/genetics/iyag026

**Published:** 2026-02-02

**Authors:** Malvika Srivastava, Ard A Louis, Nora S Martin

**Affiliations:** Institute of Integrative Biology, ETH Zurich, Zurich 8092, Switzerland; Swiss Institute of Bioinformatics, Lausanne 1015, Switzerland; Rudolf Peierls Centre for Theoretical Physics, University of Oxford, Oxford OX1 3PU, United Kingdom; CRG (Barcelona Collaboratorium for Modelling and Predictive Biology), Dr. Aiguader 88, Barcelona 08003, Spain

**Keywords:** genotype–phenotype map, fitness landscape, accessible paths

## Abstract

Ruggedness—the prevalence of fitness peaks—and navigability—the existence of fitness-increasing paths to a target—are key factors affecting evolution on fitness landscapes. Here, we analyze these properties in landscapes that inherit biophysically grounded genotype–phenotype (GP) maps. By assuming a random phenotype-fitness assignment as a baseline, the structure of the GP maps is included without imposing further fitness correlations. We show analytically that the expected ruggedness can be predicted from two quantities: the sizes of neutral components (NCs)—mutationally connected genotype sets with the same phenotype—and their evolvabilities, defined as the number of distinct phenotypes among the NC’s mutational neighbors. Other features—such as robustness—influence ruggedness only indirectly via correlations with evolvability. Numerical results across diverse GP maps confirm that NC size and evolvability alone suffice to predict both the mean prevalence and heights of peaks. These calculations also provide new insights: Under random phenotype-fitness assignment, peaks arising from high-evolvability NCs have higher expected fitness than those from low-evolvability NCs. Thus, when evolvability correlates positively with NC size, the formation of large low-fitness peaks is impeded. We further derive an approximate scaling law for the minimal average evolvability required for navigability. Our framework applies broadly across GP maps, providing general insight into when and why fitness landscapes are expected to be rugged or navigable.

## Introduction

To visualize the process of adaptation, Sewall Wright used the metaphor of the fitness landscape (Wright 1932)—wherein genotypes are represented by coordinates in a genotype space, and their fitness by the elevation of each coordinate. How an evolving population traverses a fitness landscape, is influenced by its topographical properties and by population genetic conditions. Consider the strong selection, weak mutation regime, where each new mutation is either lost or fixed in the population before the next mutation appears, causing populations to remain concentrated on one genotype at a time and to follow paths in which fitness never decreases, called *accessible paths* (de Visser and Krug 2014). These accessible paths either terminate on a sub-optimal fitness peak or on the global fitness peak—both corresponding to genotypes from which no fitness increasing mutations exist. Thus, *ruggedness* i.e. the prevalence of peaks, (Szendro et al. 2013) is an important topographical feature of fitness landscapes. Given a landscape with multiple peaks, a natural second question concerns the existence of accessible paths to high-fitness peaks (Greenbury et al. 2022; Papkou et al. 2023; Srivastava et al. 2023). Here, the global optimum is a common reference (Franke et al. 2011; Greenbury et al. 2022), and has the advantage of being identifiable in any landscape without needing additional parameters. Even in the presence of genotypes with identical fitness, the global peak is well-defined as the *set* of genotypes with the optimal fitness value (Greenbury et al. 2022). The accessibility of this global optimum is summarized by the *navigability*, the probability that an accessible path to the global optimum exists from a randomly chosen starting point (Greenbury et al. 2022).

Quantifying peaks and accessible paths in a landscape poses a challenge: to account for all peaks and accessible paths, combinatorially complete landscapes are necessary (Wu et al. 2016). These complete landscapes contain all $K^{L}$ possible genotypes and their fitness values, where *L* is the sequence length and *K* the alphabet size, and thus the scale of the landscape increases exponentially with sequence length. Therefore, computational and mathematical models, which can be scaled up easily, have a long history in the field (Kauffman and Levin 1987; Kauffman and Weinberger 1989; Aita et al. 2000). However, empirical measurements of complete landscapes of short sequence length are now also becoming possible thanks to high-throughput techniques (Blanco et al. 2019).

Besides their scalability, the strength of theoretical models lies in disentangling, how different factors shape fitness landscape topographies: for example, the House-of-Cards (HoC) model (Kingman 1978) provides a reference model, which accounts for sequence length *L* and alphabet size *K*, but is otherwise built on a random allocation of fitness values to genotypes (Kauffman and Levin 1987). Studying the role of sequence length *L* and alphabet size *K* is important because they set the *potential* (accessible and non-accessible) paths, which are central for ruggedness and navigability. For example, each genotype has (K−1)L mutational neighbors. Thus, when *L* and/or *K* is large, it becomes very likely for a genotype to have a mutational neighbor with higher fitness. Therefore, the prevalence of peaks decreases (Kauffman and Levin 1987) and their mean fitness increases (Macken and Perelson 1989) since it becomes increasingly unlikely for low-fitness genotypes to be peaks. In contrast with peaks, the existence of accessible paths in the HoC model depends primarily on the alphabet size *K* (Zagorski et al. 2016). These findings quantify the intuition that the parameters *L* and *K* are key determinants of landscape topography. Further, the HoC model provides useful context for empirical data, for instance, giving a baseline number of peaks for a landscape with a given *L* and *K* (Papkou et al. 2023).

**Fig. 1.**
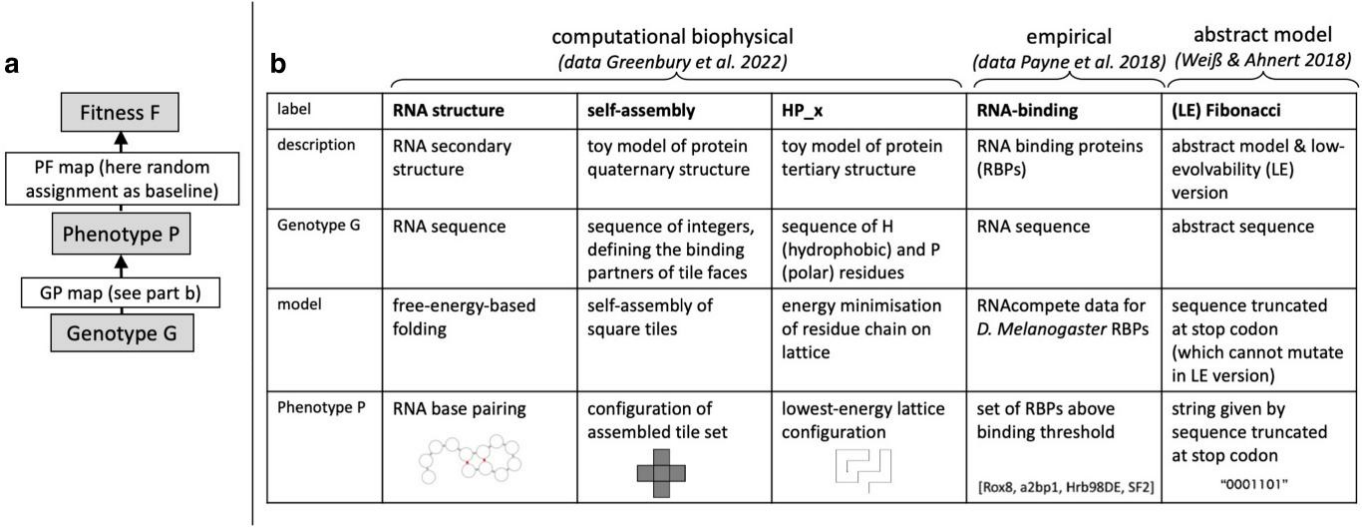
Genotype-phenotype-fitness (GPF) map construction and underlying GP maps: a) In a GPF map, each genotype is first mapped to a phenotype using a biophysical GP map, and then to fitness using a PF map, in this case, a random assignment (Rendel 2011; Greenbury et al. 2022). b) To illustrate the broad applicability of our calculations, we applied them to a range of GP maps from multiple sources (Payne et al. 2018; Weiß and Ahnert 2018; Greenbury et al. 2022). To give an overview of the underlying principles of each map, we extend and adapt the overview from Greenbury et al. (2022). The RNA drawing was made with forna (Kerpedjiev et al. 2015).

Here, we apply a similar principle to landscapes with an extra level of biological complexity: following Greenbury et al. (2022) and Rendel (2011), we start with a computational or empirical genotype–phenotype (GP) map and then assign a randomly drawn fitness value to each phenotype (see Fig. 1a), giving us a fitness landscape. Just like the HoC model is shaped by sequence length *L* and alphabet size *K*, this landscape is shaped by the structure of the underlying GP map. Taking this GP map structure into account is highly relevant because many GP map properties are found so commonly across well-studied GP maps that they are described as “universal” (Manrubia et al. 2021).

For our analysis, the most relevant shared properties of GP maps are: first, *redundancy* i.e. a given phenotype can be generated by multiple genotypes (Ahnert 2017). The redundancy of different phenotypes typically differs by orders of magnitude (Ahnert 2017). Secondly, the prevalence of neutral mutations, i.e. mutations from a genotype *g* with phenotype *p* to another genotype $g^{\prime }$ with the same phenotype *p*, is much higher than in a HoC-like, permuted version of the GP map (Greenbury et al. 2016). These two factors lead to the formation of neutral components (NCs) of different sizes (Greenbury et al. 2016): groups of genotypes that not only share a phenotype *p*, but are connected through sequences of phenotype-conserving neutral mutations (Schaper et al. 2012). As could be expected from the differences in phenotypic redundancies, NCs differ in their NC size |NC|, the number of genotypes they contain (Schaper et al. 2012). This heterogeneity in NC sizes is reflected in the mutational connections between different NCs: Larger NCs tend to have a higher number of unique phenotypes among their mutational neighbors, and this is referred to as their *evolvability*  $\epsilon_{NC}$ (Wagner 2008; Schaper et al. 2012). Since this evolvability quantifies the phenotypic diversity connected to the boundary of a *specific* NC, each NC has its own evolvability and evolvability differences within a single GP map highlight the heterogeneity in the structure of a GP map. Thus, it is important to distinguish this use of the term *evolvability* from the usage in other fields, where it is taken to describe an entire landscape (Payne and Wagner 2019). These key definitions—of NCs, NC sizes |NC| and NC evolvabilities $\epsilon_{NC}$—are summarized in Table 1.

**Table 1.** Key definitions for neutral components (NCs) - see Schaper et al. (2012).

*NC*	A set of genotypes, which share a common phenotype *p* and where any genotype can be reached from any other genotype through a series of phenotype-conserving mutations.
$\epsilon_{NC}$	An NC’s evolvability is the number of distinct phenotypes among its neighbors (2 for red NC in Fig. 2).
|NC|	An NC’s size is the number of genotypes it represents in the GP map (4 for red NC in Fig. 2).

The existence of shared structural GP map features prompts the question of how these features shape fitness landscape topographies. Analogous to how the HoC model illustrates the role of sequence length and alphabet size without assuming further correlations, building a fitness landscape from a GP map and a random phenotype-fitness (PF) map illustrates the role of GP map structure (Fig. 1a) (Greenbury et al. 2022). Prior numerical analyses have suggested that such landscapes typically have low ruggedness and high navigability, attributing this primarily to the high prevalence of neutral mutations (Greenbury et al. 2022). However, since the prevalence of neutral mutations is correlated with other GP map features, such as the sizes and evolvabilities of NCs, the exact causal and quantitative relationships remain uncharacterized.

**Fig. 2.**
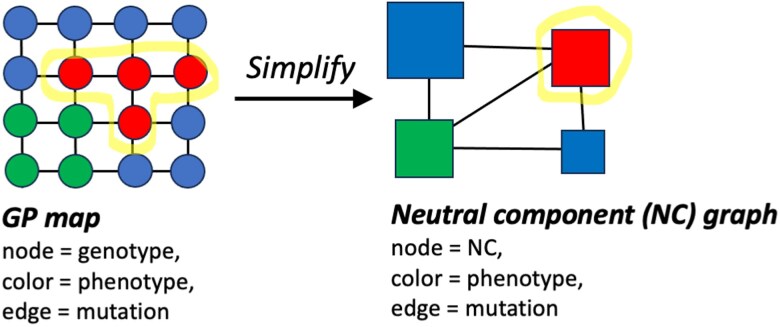
Simplifying a GP map to an NC graph: Each NC represents a set of mutationally connected genotypes that share the same phenotype (Schaper et al. 2012) and thus have a single associated fitness value. The study of ruggedness and navigability in GPF maps can be simplified by reducing the underlying GP map (left) to an NC graph (right) (Greenbury 2014), which is typically orders of magnitude smaller.

In this paper, we derive analytic predictions for the topography of such genotype–phenotype-fitness (GPF) maps. To make the analysis tractable, we work with GPF maps at the coarse-grained level of neutral components (NCs), similar to existing coarse-grained treatments (Greenbury 2014; Catalán et al. 2017; Hu et al. 2020). Mutational paths within an NC are accessible by definition, and so we can sidestep the complexity of exhaustively analyzing these paths by abstracting each NC to a single entity. This means rewriting the GP map as a network of NCs, the *NC graph*: Each NC is a node, and two nodes are connected by an edge if there is a mutational connection between the two NCs in the GP map (Greenbury 2014), see Fig. 2. Even though such an NC graph is orders of magnitude smaller than the full GP map in our examples, it contains all information needed to identify accessible paths and peaks: An NC is a peak if all its mutational neighbors on the NC graph are lower in fitness. Similarly, accessible paths can be identified from the NC graph (Greenbury 2014) since an accessible path from an initial NC to a final NC exists on the GPF map, if and only if such a path exists in the NC graph. Thus, the NC graph is ideal for our objective of characterizing landscapes topographically, including the sizes and heights of peaks and the existence of accessible paths. Other questions, such as the probability that a given path is taken in an evolutionary process, would depend on further details of the GP map and the population.

We validate our analytic predictions using simulations on a diverse set of nine GP maps (Fig. 1b). These include six biophysical computational GP maps from Greenbury et al. (2022), describing RNA secondary structure (“RNA structure”), protein quaternary structure self-assembly (“self-assembly”), and hydrophobic-polar protein folding (“HP_x,” with x indicating system size). Additionally, we build a GP map, where each genotype maps to the set of specifically binding RNA-binding proteins (note that this is a highly simplified way of defining a simple, categorical phenotype per genotype), based on empirical binding data curated in Payne et al. (2018). Lastly, to illustrate the role of evolvability, we employ an abstract GP map model (“Fibonacci”) and its low-evolvability counterpart (“LE Fibonacci”), both from Weiß and Ahnert (2018). We show that the expected prevalence of fitness peaks is completely determined by NC sizes and evolvabilities. Secondly, we find that the higher the evolvability, the higher the expected fitness of a peak, and that the expected distribution of peak heights can be approximated based on NC evolvabilities and the distribution underlying the PF map. Finally, we estimate the number of accessible paths, finding that GP maps with low average evolvability are unlikely to be navigable.

## Methods

### GP map data

All GP maps and the underlying model/data are summarized in Fig. 1; further details on data processing for the RNA-binding map, and model construction for the Fibonacci map are given in the following paragraphs. Note that all these GP maps have one qualitatively distinct *deleterious* phenotype (for example, non-terminating assemblies), which is set to zero fitness here as in previous GPF maps (Rendel 2011; Greenbury et al. 2022).

The RNA-binding map is based on a curated empirical dataset (Payne et al. 2018) of RNAcompete data for *D. Melanogaster*. To obtain a GP map with a single, categorical phenotype per genotype, we use a sequence’s *set of* specifically binding RNA binding proteins (RBPs) as a phenotype (i.e. those with an enrichment score of >0.35 as in Payne et al. 2018). Sequences without any specifically binding RBP were treated as deleterious. Note that this GP map would change if data for more RBPs were added. Further, including all RBP without focusing on specific cell types and neglecting binding strengths is a highly simplified treatment.

The Fibonacci GP map we use is Weiß and Ahnert’s (2018) generalization of the original (Greenbury and Ahnert 2015): genotypes are length-*L* strings made up of *K* distinct letters, with one letter acting as a stop codon (here L=12, K=3). For simplicity, we represent the three letters as *0*, *1* and *2*, with *2* being the stop codon. The phenotype is obtained by cutting the genotype before the first stop codon, and genotypes without stop codons are undefined/deleterious. In the low-evolvability version, the first stop codon cannot mutate (Weiß and Ahnert 2018). To ensure symmetry of allowed mutations, we additionally exclude mutations that introduce a new stop codon before the first stop codon (if *2* is the stop codon, *0202* cannot mutate to *0002*, so the reverse mutation *0002* to *0202* is also forbidden).

### NC graph construction from GP maps

First, given a GP map, we need to identify all NCs. To do so, we applied a decomposition algorithm (Grüner et al. 1996) until we had NC information for all non-deleterious genotypes (deleterious, zero-fitness genotypes play no role in accessible paths and are not included in the NC graph). Once every genotype was assigned a unique NC label, we iterated through all mutations on all genotypes to obtain all mutational connections between NCs. These pieces of information are sufficient to construct the NC graph (see Supplementary Figs. S3–S7 for a characterization of the NCs and NC graphs).

### PF maps

PF maps were set by simply listing all phenotypes in a given GP map, and drawing a single, independent fitness value for each non-deleterious phenotype from a fixed PDF, see Greenbury et al. (2022) and Rendel (2011). Where not stated otherwise, this PDF is a uniform distribution between 0 to 1, but we also include an exponential distribution with λ=1.

### Ruggedness simulations

**Fig. 3.**
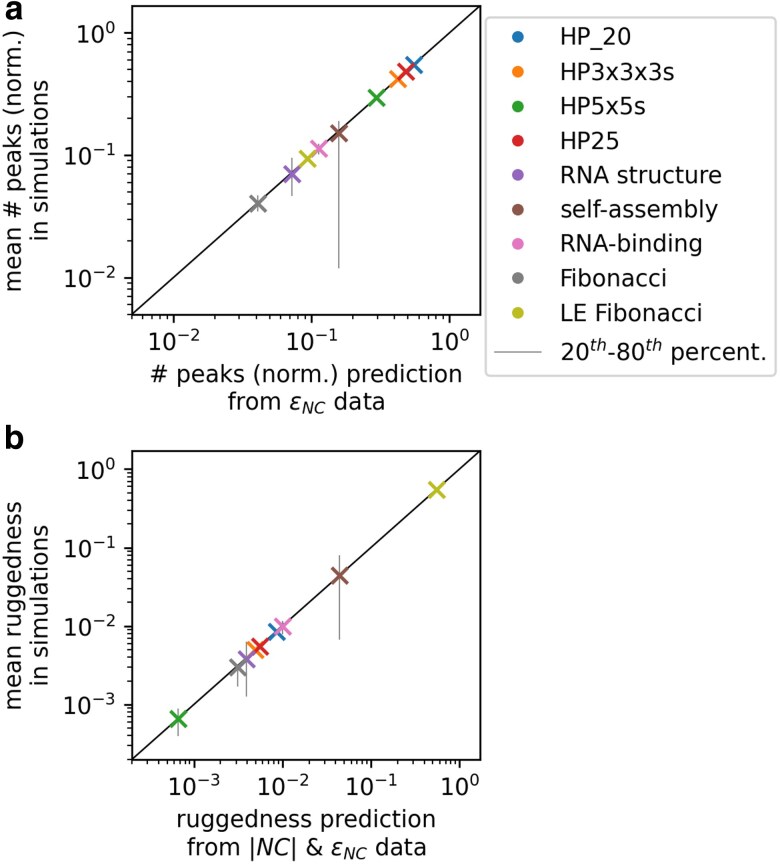
The number of peaks, and the fraction of genotypes in peaks (ruggedness), can be predicted from the set of NC sizes and evolvabilities: a) For our nine GP maps, the mean number of peaks in simulations (*y*-axis, normalized by the number of NCs in the map and averaged over $10^{3}$ PF maps) is well-predicted by equation (2) (*x*-axis), which just depends on the set of NC evolvabilities. b) Similarly, the mean ruggedness in simulations (*y*-axis, average over $10^{3}$ PF maps) is well-predicted by equation (3) (*x*-axis), which just depends on the set of NC sizes and evolvabilities. Error bars extend from the $20^{th}$ to the $80^{th}$ percentile.

**Fig. 4.**
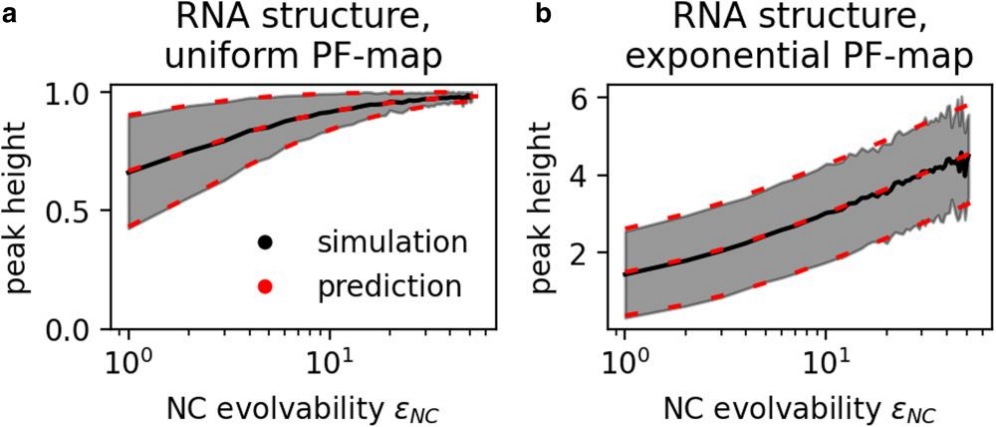
More evolvable peaks have higher expected fitness: For the simulated RNA secondary structure GPF maps, peak fitness is plotted against evolvability (mean and standard deviation in grey) for all peaks found across $10^{3}$ PF map realizations. High-evolvability peaks have higher mean fitness, in agreement with our analytic predictions (predicted mean and standard deviation as dashed red lines). a) PF map drawn from a uniform distribution (see equation (6)). b) PF map drawn from an exponential distribution (see equation (7)).

**Fig. 5.**
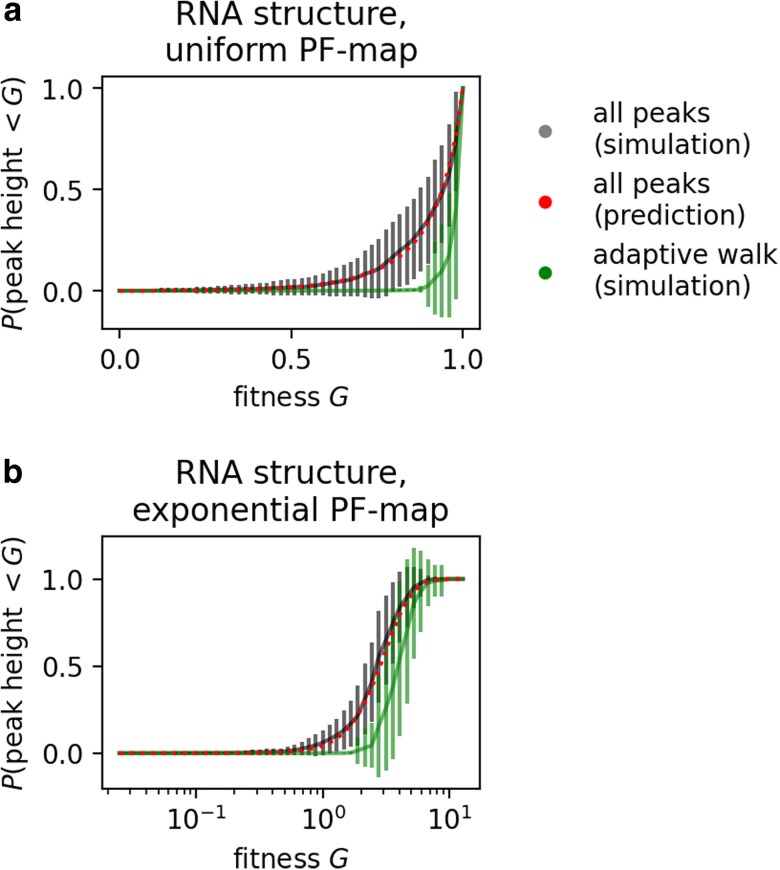
The expected peak height distribution can be approximated from NC evolvabilities and the distribution underlying the PF map: We build GPF maps from the RNA structure GP map and report the fraction of all peaks with fitness F<G (black, mean across 100 GPF maps is shown, with standard deviation as error bars). This distribution is well-approximated by equation (8) (red), which depends only on NC evolvabilities and the distribution underlying the PF map. Turning to the peaks reached by adaptive walks (green, again mean and standard deviation across 100 GPF maps), we find high peaks to be overrepresented. a) PF map drawn from a uniform distribution ($C_{F}(G)=G$ for 0≤G≤1). b) PF map drawn from an exponential distribution ($C_{F}(G)=1-\exp\;(-G)$).

For our ruggedness simulations, we generate a PF map and then set each NC’s fitness based on its phenotype. Then we identify peaks, by iterating through NCs and recording NCs whose fitness exceeds that of all neighboring NCs. Peak prevalence and ruggedness (Fig. 3 and Supplementary Figs. S2 & S8–S10) are insensitive to the choice of distribution underlying the PF map, and therefore we only show data for PF maps from uniform distributions. All other peak analyses are repeated twice, once for uniform distributions (between 0 and 1) and once for exponential distributions (λ=1). Thus, we have two sets of peak simulations, each repeated for $10^{3}$ PF map realizations per GP map. Peak prevalence and ruggedness data are based on all $10^{3}$ PF map realizations (for example, means and error bars in Fig. 3), but the more detailed analyses at the level of individual peaks only use subsets of the data: the analyses of peak heights (Fig. 4 and Supplementary Figs. S11–S14) are based on up to $5\times 10^{6}$ peaks recorded for each GP map (this is the full set of recorded peaks in some maps, for example RNA structure), and the peak height analyses at the level of individual PF maps (Fig. 5 and Supplementary Figs. S15–S20) are based on the first 100 PF maps for each GP map.

### Adaptive walks

To assess, which peaks are likely to be reached, we focus on the strong-selection-weak mutation regime and approximate the evolving population by a single adaptive walker, see de Visser and Krug (2014). This walker is modeled with the pseudo-code below (following Greenbury et al. (2022), but with non-accessible, fitness-decreasing steps excluded). We run $10^{3}$ walkers per PF map for 100 PF maps per GP map. To ensure that low-fitness peaks are not excluded merely due to the initial conditions, we initialize each adaptive walker by first listing all non-deleterious, non-peak NCs that are lower in fitness than the lowest-fitness peak. We want any genotype from these NCs to be equally likely as the starting point, and thus we first choose from these NCs with probabilities proportional to their size |NC|, and then select a genotype from the chosen NC.

**Procedure**  Adaptive walk

 $g_{0}\leftarrow$ initial genotype

 **while**  $g_{0}$ is not on a peak **do**

  l← all fitness-increasing & neutral neighbors of $g_{0}$

  P← Kimura fixation probability of each genotype in *l* (with population size $10^{3}$)

  $g_{0}\leftarrow$ select from *l*, with weights given by *P*

 **end while**

 record endpoint $g_{0}$

**end procedure**

### Navigability simulations

Each navigability value is based on 500 PF maps per GP map, and 10 randomly selected source phenotypes $p_{s}$ per PF map. For each source phenotype $p_{s}$, we perform one simulation testing for an accessible path to the global peak as follows (similar to the component network algorithm for navigability in Greenbury 2014):

- Integrating the PF map into the NC graph: We build a directed version of the NC graph, where edges point in fitness-increasing direction.
- The previous navigability definition randomly selected a starting point out of all genotypes mapping to $p_{s}$ (Greenbury et al. 2022). To replicate this on the NC graph, we list all NCs mapping to $p_{s}$, and set the probability of selecting each NC proportional to the number of genotypes it represents, i.e. its size |NC|. Then, we draw the initial NC on the path, $NC_{0}$.
- Evaluating if an accessible path exists: we use standard graph algorithms (Hagberg et al. 2008) on the directed version of the NC graph to test if a fitness-increasing, accessible path exists from $NC_{0}$ to at least one NC corresponding to the highest-fitness phenotype $p_{t}$.

Finally, we compute navigability as the fraction of accessibility simulations that were successful, i.e. where at least one path was found.

Note that this way of computing navigability differs from the original definition by Greenbury et al. (2022), where target phenotypes are first sampled and then manually set to be the global optimum. However, when averaged over PF maps, both definitions are equivalent since any fitness ranking between all phenotypes is equally likely in both cases and both cases take the global optimum as the target.

## Results

### Ruggedness is set by the sizes and evolvabilities of NCs

First, let us use the simplification of the NC graph to link the expected prevalence of peaks in a GPF map to properties of the underlying GP map. To do this, we apply existing arguments from the HoC model (Kauffman and Levin 1987) but focus on the coarse-grained level of NC graph nodes rather than individual genotypes: For an NC to be a peak, it must be fitter than its $\epsilon_{NC}$ distinct phenotypic neighbors. Upon random fitness assignment to phenotypes, any fitness ranking between the NC’s phenotype and its $\epsilon_{NC}$ neighbors is equally likely. Thus, the likelihood that an NC is a peak decreases rapidly with its evolvability as:


(1) $$P(\text{is peak})=\frac{1}{\epsilon_{NC}+1}$$

This expression is identical to that of the HoC model and derived with the same principles (Kauffman and Levin 1987), only that the relevant number of ranked fitness values is (K−1)L+1 in the HoC model and $\epsilon_{NC}+1$ in our GPF maps.

Summing over all NCs gives the expected number of peaks:


(2) $$\langle \text{number peaks}\rangle =\sum_{\mathrm{NCindexi}}\frac{1}{\epsilon_{i}+1}$$

This expression is valid due to the linearity of expectation, even though the different NCs are not independent (for example, two mutationally connected NCs cannot both be peaks under the same PF map). Thus, equation (2) is in excellent agreement with the mean number of peaks found in simulations across our nine GP maps (Fig. 3a).

However, in a GPF map, the expected *number* of peaks only tells part of the story: a GPF map with 10 peak NCs of one genotype each is not the same as a GPF map with 10 peak NCs with thousands of genotypes each. Thus, we define the *ruggedness* for a GPF map as the fraction of all *genotypes* that are in peaks. To compute this ruggedness, we need to account for each NC’s size |NC|, the number of genotypes it represents (see Fig. 2 for a schematic representation):


(3) $$\langle \text{ruggedness}\rangle =\frac{1}{K^{L}}\sum_{\mathrm{NCindexi}}\frac{\left|NC_{i}\right|}{\epsilon_{i}+1}$$

where the normalization is given by the total number of genotypes $K^{L}$. Fig. 3b demonstrates that equation (3) accurately predicts the mean ruggedness found in simulations for all nine GP map examples. Thus, for a random phenotype-fitness assignment without ties, the only factors determining the mean ruggedness are the set of NC sizes $\left\{|NC_{i}|\right\}$ and NC evolvabilities $\left\{\epsilon_{i}\right\}$. Other properties of GP maps (for example, length of the mutable sequence Greenbury et al. 2022, see SM section S2.2.1) only affect the expected prevalence of peaks indirectly insofar as they influence the NCs and their evolvabilities.

Moreover, equations (2)–(3) illustrate how the expected ruggedness follows from the structure of the GP map: higher NC evolvabilities imply a lower mean number of peaks (equation (2)), but the mean ruggedness also depends on a second factor, NC sizes (equation (3)): ruggedness will be lowest if the highest-evolvability NCs, which are least likely to be peaks, have the largest NC sizes, meaning they take up the highest fractions of genotype space. Both these factors are relevant for interpreting the data in Fig. 3: The importance of high evolvability is illustrated by the contrast between the Fibonacci GP map and the LE Fibonacci GP map, which share the same NC sizes, but differ in their evolvabilities. The LE Fibonacci map has lower evolvabilities by construction, leading to a higher mean peak count and higher mean ruggedness. Further, NC sizes are needed to understand the HP GPF maps, which all have a high mean number of peaks, but low mean ruggedness: Their large number of low-evolvability NCs leads to a high number of peaks, but these peak NCs tend to be small, due to the positive correlation between NC size and evolvability in all four HP maps (Supplementary Fig. S4): for example, zero-evolvability NCs have mean sizes of two genotypes or less in all four HP GP maps, whereas high-evolvability NCs contain tens to thousands of genotypes.

To sum up, high evolvability in a GP map, as well as a positive correlation between NC size and evolvability, lead to low expected ruggedness in the corresponding GPF map. Since this positive correlation is found across well-studied GP maps (at least at the phenotypic level Ahnert 2017, and likely also for NCs Schaper et al. 2012), including our models, the resulting GPF maps have low ruggedness, lower than in a randomized landscape of the same size (see SM section S2.2.1), with two exceptions: the LE Fibonacci model, which was specifically designed as a low-evolvability test case, and the self-assembly model, where the short sequence length chosen for computational reasons leads to a particularly low number of phenotypes (Greenbury et al. 2022) and thus limits evolvability.

### Higher-evolvability peaks tend to be higher in fitness

Given that many of our GPF maps are multi-peaked, we next turn to the height of these peaks. Again, evolvability plays a key role: High-evolvability peaks tend to have high fitness, since these NCs are only peaks if they are of higher fitness than many mutational neighbors. This can be formalized mathematically as follows: Using Bayes’ rule, we can write the probability that an NC of evolvability $\epsilon_{NC}$ has fitness *F*, given that it is a peak, as:


(4) $$P(F\;|\;\text{is peak})=P_{F}(F)\frac{P(\text{is peak}\;|\;F)}{P(\text{is peak})}$$

We have already derived P(is peak) (equation (1)), and $P_{F}(F)$ is simply the probability that the NC has fitness *F*, and thus corresponds to the PDF from which the PF map is drawn. If we denote the CDF of $P_{F}(F)$ as $C_{F}(F)$, then $P(\text{is peak}\;|\;F)=C_{F}{\left(F\right)}^{\epsilon_{NC}}$, since the focal NC is a peak if its $\epsilon_{NC}$ phenotypic neighbors are all lower in fitness than its own fitness *F*. Thus, we can write:


$$P(F\;|\;\text{is peak})=P_{F}(F)\;(1+\epsilon_{NC})\;C_{F}{\left(F\right)}^{\epsilon_{NC}}$$

As before, this mirrors an expression for HoC models, only with $\epsilon_{NC}$ replacing (K−1)L as the relevant neighborhood size (Macken and Perelson 1989). Integrating gives the following CDF:


(5) $$P_{\epsilon_{NC}}(F\leq G\;|\;\text{is peak})=C_{F}{\left(G\right)}^{\epsilon_{NC}+1}$$

This form can be recognized as the CDF of the highest order statistic, i.e. the highest value recorded after $\epsilon_{NC}+1$ independent draws from a distribution with CDF $C_{F}(G)$ (Arnold et al. 1992, p. 12). This parallel is intuitive since a peak is the maximum of itself and its $\epsilon_{NC}$ phenotypic neighbors, all with fitness values drawn from $P_{F}(F)$.

Equation (5) implies that higher-evolvability peaks tend to be of higher fitness: Since $C_{F}(G)\leq 1$, we have $P_{\epsilon_{1}}(F\leq G\;|\;\text{is peak})\leq P_{\epsilon_{2}}(F\leq G\;|\;\text{is peak})$ if $\epsilon_{1}>\epsilon_{2}$. Thus, if we compare the height distribution of peaks of two different evolvabilities, $\epsilon_{1}$ and $\epsilon_{2}$ with $\epsilon_{1}>\epsilon_{2}$, the peak height for $\epsilon_{1}$ is stochastically larger (Ross 1983, p. 251f.). Thus, the expected peak height is higher (or equal) for the higher-evolvability NC with $\epsilon_{1}$.

This derivation corresponds to the following intuition about equation (5): For any fitness below the global optimum, the CDF of all NCs is less than one ($C_{F}(G)<1$) and thus the CDF of peak heights is lower than the CDF of all NCs ($P_{\epsilon_{NC}}(F\leq G\;|\;\text{is peak})<C_{F}(G)$ for any $\epsilon_{NC}\geq 1$). Thus, low-fitness NCs are less common among the set of peaks than among the set of all (peak or non-peak) NCs. Therefore, high fitness values are overrepresented among peaks. Due to the power-law-dependence on $\epsilon_{NC}$, this effect is more pronounced for higher-evolvability NCs and thus higher-evolvability peaks tend to be of higher fitness.

While this calculation has shown that peak height tends to increase with NC evolvability for any PF map, the exact functional form depends on the distribution underlying the PF map via $C_{F}(G)$. For example, if the PF map is drawn from a uniform distribution between 0 and 1, the mean peak fitness $\langle F\rangle_{\mathrm{peak}}$ and its variance $\text{Var}(F_{\mathrm{peak}})$ can be looked up from the corresponding order theory calculations (Arnold et al. 1992, p. 14) (or derived by integration):


(6) $$\langle F\rangle_{\mathrm{peak}}=\frac{\epsilon_{NC}+1}{\epsilon_{NC}+2};\;\text{Var}(F_{\mathrm{peak}})=\frac{\epsilon_{NC}+1}{(\epsilon_{NC}+3)(\epsilon_{NC}+2{)}^{2}}$$

If the PF map is drawn from an exponential distribution with λ=1, we have (Arnold et al. 1992, p. 73):


(7) $$\langle F\rangle_{\mathrm{peak}}=\sum_{k=1}^{\epsilon_{NC}+1}\frac{1}{k}\;\;\text{Var}(F_{\mathrm{peak}})=\sum_{k=1}^{\epsilon_{NC}+1}\frac{1}{k^{2}}$$

These predictions agree with computational results (Fig. 4). In both cases, the mean peak height increases with increasing evolvability, albeit with a saturating trend for the uniform PF map, where fitness has a fixed upper limit of one.

### The peak height distribution depends on evolvabilities and the PF map

Combining the results from the previous sections, we now turn to the expected distribution of peak heights in a GPF map: equation (1) gives the probability that an NC is a peak, and, if it is a peak, equation (5) describes the probability that its height is lower than a fitness *G*. Multiplying these two expressions, summing over all NCs, and dividing by the expected number of peaks approximates the mean fraction of peaks in a GPF map with fitness lower than *G*:


(8) $$P_{\mathrm{peaks}}(F\leq G)\approx \frac{1}{\sum_{\mathrm{NCindexi}}(\epsilon_{i}+1{)}^{-1}}\sum_{\mathrm{NCindexi}}\frac{C_{F}{\left(G\right)}^{\epsilon_{i}+1}}{\epsilon_{i}+1}$$

This expression is not exact because we have normalized by the mean number of peaks, without taking fluctuations between different PF maps into account. Nevertheless, this distribution approximates the peak height distribution in simulated GPF maps well, as shown in Fig. 5 for the RNA secondary structure map as an example, and in Supplementary Figs. S17 & S18 for the other GP maps.

Now, with the distribution of all peak heights in a GPF map as a baseline, we turn to the fitness reached at the end of an adaptive walk, which steps from a genotype to a neutral or fitter mutational neighbor until a peak is reached (see methods section). Among the endpoints of these simulated walks, low-fitness peaks are under-represented compared to their share of all peaks in the GPF map (Fig. 5). Thus, higher-fitness peaks are more likely to be the endpoint of adaptive walks than the low-fitness peaks in the same landscape, and this could have several causes: First, low-fitness peaks typically have low evolvability and are therefore less connected on the NC graph, which could make them less likely to be reached. Moreover, low-evolvability NCs tend to have small NC sizes, and thus may be disfavored by the “arrival of the frequent” effect (Schaper and Louis 2014), whereby transitions to larger NCs can occur from a higher number of genotypes and are thus more likely to happen first. Both of these factors, low evolvability and small NC size of low-fitness peaks, are rooted in the heterogeneity of the underlying GP map. In addition to these GP-map-specific factors, there could be further reasons why higher peaks are more likely to be reached: even in the standard HoC landscape, i.e. without any differences in NC evolvabilities or sizes, higher-fitness peaks are more likely to be reached (Macken and Perelson 1989).

### Minimum evolvability required for navigability in simple GP maps

Given that many GPF maps are multi-peaked, a second key question is whether they nevertheless have fitness-increasing, i.e. accessible, paths to a target. For GPF maps specifically, the *navigability* lets us quantify whether GPF maps built from a given GP map are likely to have such accessible paths (Greenbury et al. 2022). Choose a random source phenotype $p_{s}$ and start from a random genotype $g_{0}$ mapping to this $p_{s}$. Then, the *navigability* is the probability that an accessible path exists from $g_{0}$ to any genotype producing the highest-fitness phenotype $p_{t}$, i.e. the global optimum of the landscape, averaged across source phenotypes $p_{s}$ and PF maps (Greenbury et al. 2022).

While navigability is harder to predict from simple NC characteristics than ruggedness, we can make progress for a simple case: GP maps with a single NC per phenotype, where each NC connects to any of the $n_{p}$ phenotypes with a constant probability ${\bar{\epsilon }}_{NC}/(n_{p}-1)$. In this scenario, an NC graph is expected to be non-navigable, with a navigability lower than *δ*, if (see approximate calculations in SM section S1.1 and Supplementary Fig. S1):


(9) $$\epsilon_{NC}⪅n_{p}\;\left({\left(\delta \;n_{p}\right)}^{\frac{1}{n_{p}}}-1\right)$$

Thus, a highly connected, high-evolvability NC graph is a minimum requirement for the existence of accessible paths, as hypothesized by Greenbury (2014). This calculation is based on the scaling of the expected number of accessible path—if the expected number is less than *δ*, then at most one in 1/δ cases can have an accessible path (Schmiegelt and Krug 2014). This scaling does not answer the reverse question, whether high-evolvability GP maps have high navigability, since further information would be needed, for example the variance in the number of paths (Schmiegelt and Krug 2014): A high expected number of paths is consistent with both high and low navigability, since there could be a few paths per PF map (i.e. high navigability), or no paths for most PF maps and ≫1 paths for a small number of PF maps (i.e. low navigability). Obtaining this additional information is complex, even for structured graphs such as tree graphs (Nowak and Krug 2013). However, even without this information, it is clear that any GPF map will become navigable in the maximum-evolvability limit $\epsilon \to n_{p}$ since single-step paths from a source to a target become possible and these are always accessible.

It is important to emphasize the caveats in this derivation, most importantly: First, the calculations assume that any NC is equally likely to be connected to any other NC, but real NCs may be more likely to be connected to other NCs that are close in genotype space (although NCs can span the maximum genotypic distance in the GP map Schuster et al. 1994). Second, the number of NC transitions on an accessible path is assumed to be small compared to the number of phenotypes, given the high number of phenotypes in large, realistic GP maps. Third, the calculations assume a single NC per phenotype. Fourth, the NC graph is assumed to be characterized by a single evolvability ϵ, ignoring the heterogeneity of GP maps. Due to these caveats, the most important takeaway from this calculation may not be the exact quantitative bound in equation (9), but the qualitative result that low-evolvability NC graphs are non-navigable, as hypothesized in Greenbury (2014).

Despite these caveats, we apply equation (9) to our GPF maps to test if maps falling well within the inequality are indeed of low navigability. To do so, we need to make adjustments to deal with the third and fourth caveats: Our GP maps typically have multiple NCs per phenotype (like the blue phenotype in the schematic in Fig. 2). To resolve this, we build a more connected version of each GP map, where all NCs of a phenotype are merged to a single NC through additional mutational links. If the bound from equation (9) is not even met by evolvabilities derived from this *more* connected version of a given NC graph, the GP map is unlikely to be navigable. To address the next discrepancy—that different NCs in a single GP map have different evolvabilities—we characterize each map by the geometric mean of all its NC evolvabilities (treating zero evolvability values as 0.01, to avoid a single zero-evolvability NC setting the entire mean to zero). The choice of the geometric mean was motivated by the fact that evolvabilities enter the calculation as a product. However, on a logarithmic scale, the geometric and arithmetic mean and median evolvabilities of our GP maps are highly correlated (SI Supplementary Fig. S5), suggesting that they capture similar information and the choice of the geometric mean does not strongly affect the plotted results.

**Fig. 6.**
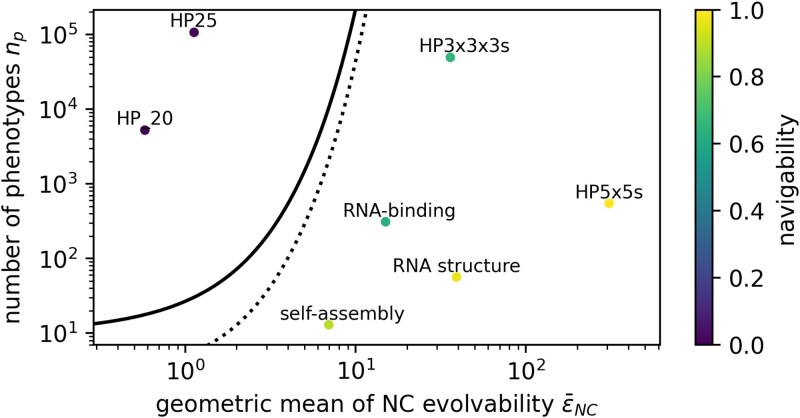
Evolvability plays a key role for navigability: Equation (9) (black and dashed line, using thresholds of δ=0.1 and δ=0.5) successfully identifies an evolvability below which our GP maps are non-navigable, despite relying on strong approximations: For each GP map, the number of phenotypes is plotted against the geometric mean of its evolvabilities (since a single $\epsilon_{NC}=0$ would bring the geometric mean to zero, this is treated as $\epsilon_{NC}=0.01$), with navigability shown on the color scale. To obtain a single evolvability value per phenotype, we use a more navigable version of each map as a basis for the evolvability data, where all NCs of a single phenotype are mutationally connected—if even this version is non-navigable, the original would be non-navigable. The Fibonacci model is excluded and analyzed in SM section S2.5.2.

Figure 6 shows that even with these simplifications and despite relying on strong approximations, equation (9) successfully identifies a low-evolvability regime in which our non-navigable GP maps fall: the two GP maps with evolvabilities clearly below the bound from equation (9) are of low navigability. Note that only the evolvability values on the *x*-axis rely on the simplifications outlined above; the navigabilities are computed numerically on the full NC graphs (see Methods section). Further applications of equation (9) to larger sets of GP maps based on the Fibonacci model as well as on synthetic NC graphs with different evolvability distributions, are given in Supplementary Figs. S21–S23.

## Discussion

To sum up, we focus on fitness landscapes built from GP maps with a random phenotype-fitness map and make analytic predictions about their topographical properties: First, the mean ruggedness only depends on the NC sizes and evolvabilities, with other GP map properties having indirect effects through their influence on these two characteristics of NCs. Further, the prevalence of peaks in GPF maps decreases when NC size and evolvability are positively correlated, which holds across well-studied GP maps (at least for phenotypic evolvability Wagner 2008; Ahnert 2017, and likely also for NCs Schaper et al. 2012). Secondly, high-evolvability NCs, if they form peaks, have exceptionally high fitness. These high-evolvability NCs tend to be large (Wagner 2008; Schaper et al. 2012) and may thus be more likely to arise as potential variation in evolutionary processes (Schaper and Louis 2014). Finally, we turned to accessible paths and derived a scaling that identifies high evolvability as a minimum requirement for navigability, and, despite relying on strong approximations, successfully identifies non-navigable GP maps in our dataset. With the exception of peak heights and adaptive walks, our results only depend on the fitness rank of different phenotypes and thus the distribution underlying the phenotype-fitness map is not important, as long as the phenotype-fitness assignment is set by independent random draws.

While we used the strictest possible interpretation for the term *neutral*, applying the term only to genotypes with identical phenotypes, this assumption could be relaxed by changing the phenotypic representation in the GP map. For example, previous work on the RNA secondary structure GP map contrasted two versions of the map: one with the full base pairing information as the phenotype and one with a more coarse-grained topological treatment of the structure as the phenotype (Mohanty et al. 2023). Once a GP map with the appropriate phenotypic information is set up, the analysis from this paper can be applied to this GP map.

Since a positive correlation between NC size and evolvability is one key factor for low ruggedness and for suppressing large, low-fitness peaks, the roots of this correlation are highly relevant to this study: Although all genotypes in an NC generate the same phenotype, high evolvability requires that these genotypes have different alternative phenotypes in their mutational neighborhoods, a point first made by Weiß and Ahnert (2018). Shifts in mutational effects due to genetic background are known as epistasis and thus we will call this specific form *evolvability-enhancing epistasis* (note that this terminology differs from Weiß and Ahnert 2018). The significance of evolvability-enhancing epistasis is illustrated by the LE Fibonacci map, which lacks evolvability-enhancing epistasis by construction (Weiß and Ahnert 2018) and has a much higher ruggedness than the standard Fibonacci map. Besides this evolvability-enhancing epistasis, however, other types of epistasis in GP maps are likely to increase ruggedness: for example, epistasis can break NCs into smaller, lower-evolvability units (Schaper et al. 2012). Even so, the fact that evolvability-enhancing epistasis can lower ruggedness, for example in the Fibonacci model, may seem at odds with the intuition that epistasis *increases* ruggedness: for example, the prevalence of sign epistasis is correlated with the prevalence of peaks (Szendro et al. 2013). However, there are existing examples of epistasis facilitating evolutionary paths, by lowering the number of nonfunctional genotypes (Metzger et al. 2024), or in the context of a funnelled landscape (Schulz et al. 2025). Moreover, evolvability-enhancing epistasis differs from metrics like the prevalence of sign epistasis in that it focuses on the GP map only. This is a useful choice for our goal of linking GP map characteristics to the resulting GPF map topographies but does not directly correspond to established metrics of epistasis in fitness landscapes. Future work should investigate the connection between these different facets of epistasis more thoroughly.

Due to the parallels between our random phenotype-fitness map, and the random *genotype*-fitness map in HoC models, it is instructive to contrast the calculations in the two cases: for example, the probability that a random genotype is a peak is 1/((K−1)L+1) in the HoC model (Kauffman and Levin 1987), and the probability that a random NC is a peak is $1/(\epsilon_{NC}+1)$ in our GPF maps. These expressions are the same, only that the relevant neighborhood size is (K−1)L in the HoC model and $\epsilon_{NC}$ in our GPF maps. Further parallels exist, for example for expected peak heights, which increase with the neighborhood size in the HoC model (Macken and Perelson 1989) and with $\epsilon_{NC}$ in our GPF maps. However, there is one key difference: Without a GP map, the mutational neighborhood size (K−1)L is usually the same for all genotypes, but in GPF maps, the relevant mutational neighborhood size is the evolvability ϵ, which can vary by orders of magnitude *within* a single GP map (see Supplementary Fig. S4), and is correlated with further features like NC sizes. Thus, the heterogeneity in the underlying GP map leads to a richer phenomenology, with NCs having vastly different sizes and evolvabilities, and thus different probabilities of being peaks and expected fitness if they are peaks. This heterogeneity can be highly relevant not only for the topography, but also for evolutionary processes: for example, in our GPF maps, low-fitness peaks tend to be small, which could make them less likely to be reached in adaptive evolution (Schaper and Louis 2014). If these NC-size effects play an important role for evolutionary outcomes, then GPF maps may add a qualitatively new contribution to a recent discussion of “rugged yet […] navigable” landscapes (Papkou et al. 2023; Westmann et al. 2024; Li and Zhang 2025): multi-peaked landscapes, in which high-fitness peaks are overrepresented among the endpoints of adaptive walks.

Our focus on random PF maps represents an analytically tractable baseline scenario in the same way that existing non-GP-map-based fitness landscape models like the HoC model represent idealized cases. Such a simple baseline is important for two reasons: first, realistic PF maps will be highly system-specific. Secondly, simple PF maps, like the random one used here, allow us to systematically disentangle the role of various factors (GP map structure, PF map structure etc.). Future work should complement our analysis with the opposite limiting case of a perfectly correlated PF map, where fitness depends linearly on the difference between the current phenotype and a fixed target phenotype—see Greenbury et al. (2022). Because adjacent NCs tend to be phenotypically similar (Dingle et al. 2025; Garcia-Galindo and Ahnert 2025), correlated PF maps introduce additional fitness correlations. Thus, high-fitness NCs are more likely to have other high-fitness NCs as neighbors, potentially reducing their likelihood of being peaks and creating a less rugged landscape. This reasoning agrees with numerical results, showing that correlated GPF maps are more navigable (Greenbury et al. 2022). However, these numerical results have to be interpreted with one confounding factor in mind: since distance to the target is often discrete (for example, base pairing distance for RNA secondary structure), multiple NCs in correlated landscapes have the same fitness, creating multi-NC fitness plateaus, which could also lower ruggedness and increase navigability. Future work should thus investigate the causal and quantitative links carefully, building on the methods and results used in the current paper: for example, NCs with evolvability $\epsilon_{NC}=1$ are peaks unless their single phenotypic neighbor happens to be phenotypically more similar to the target, and thus evolvability is likely to keep a key role in shaping peak identities and heights. Such research, both for single-target and multi-target PF maps, would complement our random PF maps and form a basis for more realistic and more complex PF maps.

A second topic for future work is to go beyond the topographical description of our landscapes to a probabilistic one, describing the population genetics on this landscape. Our adaptive walk simulations suggest that small, low-fitness peaks are less likely to be reached in the strong-selection-weak-mutation regime. This could have several explanations, including differences in NC sizes and evolvabilities set by the underlying GP map. Developing theory to disentangle these factors is an interesting topic for future work, including more detailed and realistic simulations of evolving populations to assess, how outcomes depend on population-genetic assumptions like the strong-selection-weak-mutation regime and population size. To build a theoretical basis, this research could construct more complex versions of NC graphs, which include information on the number of mutations connecting two NCs as edge weights, similar to the weighted graphs by Hu et al. (2020) and Catalán et al. (2017). In addition, the GPF maps could further be used to investigate more general open questions about evolution on complex fitness landscapes, for example whether populations evolve toward certain local topographies (see Payne and Wagner 2019).

Finally, the effectiveness of using the NC graph for this analysis suggests it could help provide useful quantitative insights into other aspects of GP map structure and their evolutionary consequences.

## Supplementary Material

iyag026_Supplementary_Data

## Data Availability

Our code is available at https://github.com/noramartin/gpf_maps/. Data underlying the GP maps: the biophysical GP maps (Greenbury et al. 2022) are read in from Greenbury et al. 2022: https://github.com/sgreenbury/gp-maps-nav/tree/release/gp_maps; the RBP data (Payne et al. 2018) was provided by J. L. Payne and is available in our GitHub repository; the Fibonacci model (Weiß and Ahnert 2018) is re-implemented in our code. Supplemental material available at GENETICS online.
