# Supplementary material for: Predicting the topography of fitness landscapes from the structure of genotype–phenotype maps
Source: Genetics. 2026 Feb 2;232(4):iyag026. doi: 10.1093/genetics/iyag026 (PMC13050206; doi:10.1093/genetics/iyag026)
Supplement: iyag026_Supplementary_Data [file iyag026_supplementary_data.pdf]

Supplementary Information: Predicting the topography of fitness landscapes from the structure of genotype-phenotype maps

Malvika Srivastava, Ard A. Louis, Nora S. Martin

Contents

S1 Analytic derivations **1**
S1.1 Scaling of minimum evolvability $\bar{\epsilon}_{NC}$ required for navigability 1
S1.2 Analytic expression for ruggedness in the Fibonacci model 5
S1.3 Extrapolating ruggedness to large GP maps 6
S1.3.1 Scaling in HoC models as a baseline 6
S1.3.2 Scaling in GP maps 7

S2 Supplementary computational analyses **12**
S2.1 Additional NC graph analyses 12
S2.1.1 Number, evolvabilities and sizes of NCs 12
S2.1.2 Treatment of evolvabilities for main text Fig.6 14
S2.1.3 NC graph network analysis 15
S2.2 Additional ruggedness analyses 16
S2.2.1 Changes to the underlying GP maps, including dimensionality and correlations 16
S2.2.2 The impact of deleterious phenotypes and zero-evolvability NCs on ruggedness 18
S2.3 Additional analyses on the expected height of a given peak of evolvability ϵ 20
S2.4 Additional analyses on peak height distributions 24
S2.5 Additional navigability analyses 26
S2.5.1 Navigable vs. non-navigable synthetic NC graphs 26
S2.5.2 Navigable vs. non-navigable GP maps in a sample of GP maps based on the Fibonacci model 29
S2.5.3 Disjoint components and navigability in NC graphs 30

S1 Analytic derivations

S1.1 Scaling of minimum evolvability $\bar{\epsilon}_{NC}$ required for navigability

Navigability is a continuous quantity characterising a GP map: it captures the probability that there is at least one fitness-increasing (i.e. accessible) path from a randomly chosen initial phenotype to the global maximum as a target phenotype [1]. Here, we only focus on distinguishing non-navigable GP maps (with

navigability ≈ 0) from potentially navigable ones. Since accessible paths can only exist on a GP map if they exist on the corresponding NC graph, we will focus on the number of accessible paths on a NC graph.

To simplify the calculations, we will assume that the GP map has one NC per phenotype. Then, each NC has a unique fitness value, and its degree equals its evolvability. Further, we assume that the NC graph's degree distribution is narrow and can be characterised by a single evolvability value ϵ_{NC}. With these two simplifications, we estimate the number of accessible k-step paths on the NC graph:

1. **Combinatorics of fitness-increasing length-k sequences of NCs:** A length-k path involves the source, target and $k-1$ intermediate nodes. If the NC graph was a *complete graph* (i.e. with an edge between any two nodes), then these $k-1$ intermediate nodes could be any of the $n_p - 2$ nodes of the graph other than the source or target. Since these $k-1$ nodes can only be traversed in order of increasing fitness, their order along the path is fixed, and there are $\binom{n_p-2}{k-1}$ fitness-increasing sequences of intermediate nodes.

2. **Connectivity of the NC graph and source fitness**: Next, we correct for the fact that the NC graph is not complete and that some NCs are lower in fitness than the source and thus cannot appear as intermediate nodes.

   - For each step on the path, we approximate the probability that this step is mutationally possible by $\frac{\epsilon_{NC}}{n_p-1}$ since our simplifying assumption is that each node is connected to ϵ_{NC} out of $n_p - 1$ NCs. Note that this treatment further assumes that each NC is equally likely to be connected to any other NC.
   - Further, only NCs fitter than the source and less fit than the target can appear as intermediate nodes. While navigability is typically defined with the global peak as a target [1], such that all NCs are automatically less fit than the target, we develop our calculations for a more general peak. If we assume a uniform fitness distribution[1], the source has a fitness $1-\beta$ and the target a fitness of $1-\alpha$ (with $\beta > \alpha$), then the probability that all selected intermediate nodes have fitness higher than the source and lower than the target is $\approx (\beta - \alpha)^{k-1}$. This approximate treatment assumes $k \ll n_p$, such that each intermediate node is an independent draw from a larger set of NCs and fitness values. For the global optimum, we would set $\alpha = 0$ since there are no higher-fitness phenotypes to be excluded by definition.

Then, we apply the principle of linearity of expectation, and approximate the number of accessible k-step paths as:

$$\langle \text{number of k-step paths} \rangle (\beta) \approx \binom{n_p-2}{k-1} \left(\frac{\epsilon_{NC}}{n_p-1} \right)^k (\beta - \alpha)^{k-1}$$

Since we are interested in the *average* number of paths over all PF maps, we first take the mean over β, which characterises the source fitness ($F_{\text{source}} = 1 - \beta$) and is uniformly distributed between zero and the

[1] Since accessible paths only depend on fitness rankings, the result should not depend on the fitness distribution as long as it remains continuous.

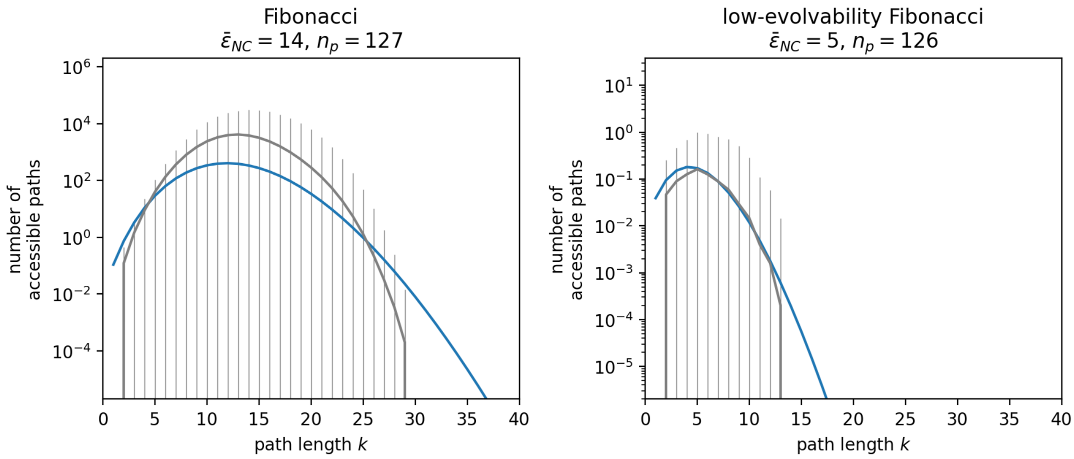

Figure S1: **Average number of accessible paths of length k in the NC graph of the Fibonacci model and its low-evolvability version:** here, we test an intermediate result of our navigability calculations (eq. (1), blue line): the number of accessible k-step paths from a source to a target on a NC graph. The grey curves represent simulation averages (with standard deviations). We see clear differences between the Fibonacci GP map on the left and its low-evolvability version on the right (note the different y-axis scales). These differences are well-approximated by the estimate from eq. (1), based only on two GP map parameters: $\bar{\epsilon}_{NC}$ and n_p. Parameters: average and standard deviation computed from 5000 simulations: 500 PF maps & 10 source phenotypes for each PF map; the target is the highest-fitness phenotype in the PF map (i.e. $\alpha = 0$). Sequence length $L = 7$ & alphabet size $K = 3$ (note that this GP map is very small for reasons of computational feasibility). The zero-evolvability phenotype in the LE version is discarded from the NC graph.

target fitness α:

$$\begin{aligned}\langle \text{number of k-step paths} \rangle &\approx \binom{n_p - 2}{k - 1} \left(\frac{\epsilon_{NC}}{n_p - 1}\right)^k \int_\alpha^1 d\beta \cdot (\beta - \alpha)^{k-1} \\ &\approx \binom{n_p - 2}{k - 1} \left(\frac{\epsilon_{NC}}{n_p - 1}\right)^k \times \frac{(1-\alpha)^k}{k} \qquad (1)\end{aligned}$$

To test this intermediate result against simulation data, we applied it to the Fibonacci model and its low-evolvability version (Fig. S1). We focus on accessible paths to the global peak ($\alpha = 0$) since our main goal is to put bounds on navigability, which is defined relative to the global peak. To apply the calculations to the two example NC graphs, we need to find a single representative ϵ_{NC} from the full distribution of ϵ_{NC} on each NC graph. Since ϵ_{NC} enters the calculation as a product, we used the geometric mean $\bar{\epsilon}_{NC}$ of all ϵ_{NC} values. We find that, despite this oversimplified treatment of the degree distribution, the simple estimate from eq. (1) approximates the order of magnitude, as well as the contrast between the Fibonacci model and its low-evolvability version well.

Having approximated the number of length-k accessible paths on the NC graph, we can sum over k to

estimate the total number of paths:

$$\langle\text{number of paths}\rangle \approx \sum_{k=1}^{n_p-1} \binom{n_p-2}{k-1} \left(\frac{(1-\alpha)\ \epsilon_{NC}}{n_p-1}\right)^k \times \frac{1}{k}$$

For simplicity, let us denote $N = n_p - 1$ and $c = \frac{(1-\alpha)\ \epsilon_{NC}}{n_p-1}$. Then we have

$$\langle\text{number of paths}\rangle \approx \sum_{k=1}^{N} \binom{N-1}{k-1} c^k \times \frac{1}{k}$$

Using the binomial identity $\binom{N-1}{k-1} = \frac{k}{N}\binom{N}{k}$:

$$\begin{aligned}\langle\text{number of paths}\rangle &\approx \frac{1}{N}\sum_{k=1}^{N} \binom{N}{k} c^k \\ &\approx \frac{1}{N}\left((1+c)^N - 1\right) \end{aligned} \tag{2}$$

Note that eq. (2) estimates the mean number of accessible paths *on the NC graph.* While the number of paths in genotype space can be much higher due to neutral mutations, each accessible path on the NC graph corresponds to at least one accessible path in genotype space. Thus, we can use the fact that the navigability is bounded by $P(\text{at least one path exists}) \leq \langle\text{number of paths}\rangle$ [2], to find that a NC graph will be non-navigable (navigability $< \delta$ for a small number δ) if:

$$\delta \gtrapprox \frac{(c+1)^N - 1}{N}$$

Since we are interested in the case $n_p \gg 1$, we have $N \approx n_p$ and $1/N \ll 1$. Then we can simplify to:

$$\begin{aligned}\delta &\gtrapprox \frac{(c+1)^{n_p}}{n_p} \\ c &\lessapprox (\delta\ n_p)^{\frac{1}{n_p}} - 1\end{aligned}$$

And thus, substituting $c = \frac{(1-\alpha)\ \epsilon_{NC}}{n_p-1} \approx \frac{(1-\alpha)\epsilon_{NC}}{n_p}$:

$$(1-\alpha)\epsilon_{NC} \lessapprox n_p\ \left((\delta\ n_p)^{\frac{1}{n_p}} - 1\right)$$

Due to the term $(1-\alpha)$, the inequality is much more likely to hold for low-fitness peaks with $\alpha \approx 1$, illustrating the following intuitive argument: if the fitness of the target is low (relative to all fitness values), then fewer GP maps have accessible paths to this target, even from a starting NC that is lower in fitness than the target. This lower accessibility results from the small number of NCs that are lower-fitness than the target and can serve as intermediate steps on accessible paths to the target.

Our main interest is in navigability, which is defined with respect to the global peak with $\alpha = 0$. Then we expect a GP map to be non-navigable if:

$$\epsilon_{NC} \lessapprox n_p\ \left((\delta\ n_p)^{\frac{1}{n_p}} - 1\right) \tag{3}$$

Thus, low-evolvability GP maps, with evolvabilities below the bound in eq. (3) are expected to have low navigability.

An interesting question is whether the reverse statement also holds, i.e. that high-evolvability GP maps have high navigability. To put such a lower bound on navigability, we would need to know not only the expected number of paths, but also its variance for the following reason [2]: NC graphs with a high expected number of paths could nevertheless be of low navigability if there were no paths for most PF maps and $\gg 1$ paths for a small number of PF maps. Computing this variance is an interesting topic for future work (similar to work on tree graphs [3]). However, even without computing the variance, it is clear that a GP map will become navigable in the maximum-evolvability limit $\epsilon \to n_p$, since single-step paths from a source to a target become possible and these are always accessible.

It is important to emphasise the caveats in this derivation, especially: (1) It assumes that the network is characterised by a single degree ϵ_{NC}, which is not true for real NC graphs. In applications of eq. (3) (see section S2.5), we will take the geometric mean $\bar{\epsilon}_{NC}$ as a proxy[2]. (2) The calculations assume that any NC is equally likely to be connected to any other NC, but real NCs may be more likely to be connected to other NCs that are close in genotype space (although some NCs can span the maximum genotypic distance in the GP map [4]). (3) The combinatoric terms assume a single NC per phenotype. (4) The fitness of the source phenotype is accounted for in a highly simplified way via the parameter β, which effectively assumes $k \ll n_p$, i.e. that path lengths are short compared to the number of phenotypes.

Due to these caveats, the most important takeaway from this calculation may not be the exact quantitative bound in eq. (3), but the qualitative result that low-evolvability NC graphs are non-navigable, as hypothesised in [5], and that this bound also depends on combinatoric terms, here the number of phenotypes n_p.

S1.2 Analytic expression for ruggedness in the Fibonacci model

One advantage of the Fibonacci model is that its GP map characteristics, such as evolvability, can be calculated analytically as parametric equations of a single parameter l, the number of sites before the first stop codon [6][3]. Since each phenotype corresponds to a single NC in the Fibonacci model, NC evolvabilities and sizes are equivalent to phenotypic evolvabilities and sizes, which are given by the following parametric equations [6]:

$$|NC|(l) = K^{L-l-1} \tag{4}$$

$$\epsilon_{NC}(l) = (K-1) \times l + (K-1) \times \left(\sum_{i=0}^{L-l-2} (K-1)^i \right) \tag{5}$$

There are S NCs with evolvability $\epsilon_{NC}(l)$ and size $|NC|(l)$, where $S(l)$ is given by [6]:

$$S(l) = (K-1)^l$$

In the low-evolvability reference model, the sizes $|NC|$ and numbers S of NCs are unchanged; only the evolvability has to be re-calculated. Since in our version of the low-evolvability model, we only allow (K-2) mutations per site before the stop codon, the evolvability in the low-evolvability model is $\epsilon_{NC}(l) = (K-2) \times l$,

[2]Since a single zero-evolvability node turns the geometric mean to zero, we treated zero evolvability values as 0.01

[3]To simplify expressions, we use a slightly different notation than the original paper, which included the stop codon in l [6]. Further, we ignore mutations to the deleterious phenotype with no stop codon.

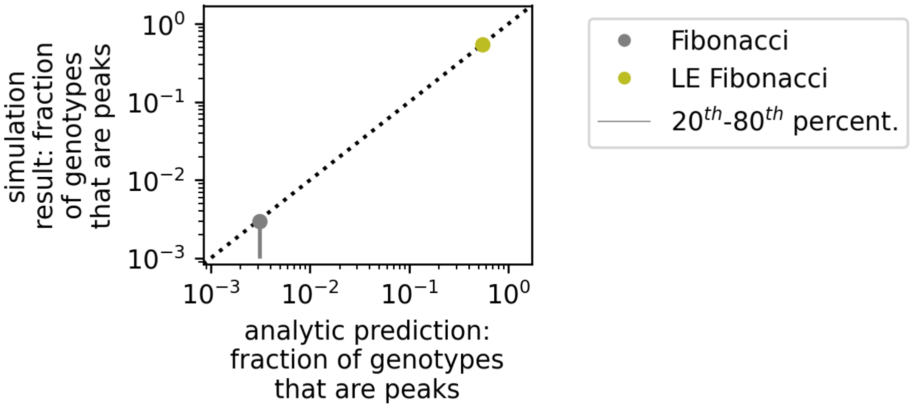

Figure S2: **Analytic ruggedness calculations for the Fibonacci model:** in the Fibonacci model, NC evolvabilities ϵ_{NC} and NC sizes $|NC|$ can be computed analytically, meaning that the mean ruggedness can be predicted purely analytically (eq. (6)), without relying on any computationally derived GP map properties. Here, simulation data (y-axis; mean is shown with 20th and 80th percentile; data from main text Fig. 3) is plotted against the purely analytic prediction (x-axis, eq. (6)). Parameters: sequence length $L = 12$, alphabet size $K = 3$, simulated ruggedness based on 10^3 sampled PF maps.

rather than $(K-1) \times l$ as in the original formulation of the model [6].

With this, we can compute the ruggedness analytically rather than relying on the computational data for evolvabilities and NC sizes:

$$\langle \text{fraction of genotypes in peaks} \rangle = \frac{1}{K^L} \sum_{l=0}^{L-1} \frac{S(l) \times |NC|(l)}{\epsilon_{NC}(l) + 1} \tag{6}$$

This analytic prediction is shown in Fig. S2 and is in excellent agreement with the simulation data for both the standard and low-evolvability Fibonacci models.

S1.3 Extrapolating ruggedness to large GP maps

The sequence lengths of the GP maps studied in this paper are limited by computational feasibility. More realistic GP maps would have longer sequence lengths L, and thus a higher number of genotypes $N_G = K^L$, a higher number of phenotypes n_p and a higher number of NCs n_c. To estimate how ruggedness might scale if we studied larger GP maps, we need to make analytic extrapolations.

S1.3.1 Scaling in HoC models as a baseline

As a baseline for our extrapolations, we take a classic fitness landscape baseline, the *House-of-cards* (HoC) landscape [7, 8], where each genotype maps to a random fitness value. Since each genotype's fitness corresponds to an independent draw from a continuous distribution, genotypes do not have neutral neighbours and form single-genotype NCs. Since each single-genotype NC has $(K-1)L$ mutational neighbours, we can

define a HoC-evolvability of $\epsilon_{\text{HoC}} = (K-1)L$ distinct fitness values around each NC, giving a ruggedness of $R_{\text{HoC}} = \frac{1}{(K-1)L+1} \approx \frac{1}{(K-1)L}$ [8]. We could also introduce deleterious genotypes into the HoC model, to build a more accurate baseline for our GP maps. Similar to the deleterious phenotypes in our GPF maps, where deleterious phenotypes represent outcomes such as non-terminating assemblies, these deleterious genotypes would have zero fitness and thus cannot form part of accessible paths. This would reduce the HoC-evolvability by a factor that depends on the fraction of deleterious genotypes, and increase the HoC-ruggedness.

S1.3.2 Scaling in GP maps

The analogous calculation for GPF maps is more complex because genotypes mapping to the same phenotype do form large connected neutral components (NCs) [9]. Thus, we cannot treat each genotype separately and need to estimate how NC sizes and evolvabilities scale with L. Here we will work with a normalised version of a NC's size, the *NC frequency* f_r, which equals the size of a neutral component, normalised by the total number of genotypes in the GP map $f_r = |NC_r|/K^L$. The index r denotes the rank, i.e. the number of NCs with larger or equal size (see Fig. S3).

S1.3.2.1 NC frequency scaling Since the distribution of phenotypic frequencies is highly biased in GP maps [10–12], we also expect the NC frequency distribution to be biased. Based on our computational data in Fig. S3, we will work with a simple power-law ansatz:

$$f_r = A/r^m \tag{7}$$

Here, the rank r, the number of NCs of equal or higher size, can go from $r = 1$ for the largest NC to $r = n_c$ for the smallest NC (where n_c is the total number of NCs in the GP map).
The use of power laws is also supported by theoretical arguments based on constrained and unconstrained sites [13], which suggest a power law for *phenotypic* frequencies, which may translate to a power law in *NC* frequencies. Note, however, that these arguments can also predict other functional forms [14], including the log-normal/binomial distributions found computationally for the RNA secondary structure GP map [15].

The parameter A in eq. (7) follows from the normalisation $1 = \sum_r f_r \approx \int_{r=1}^{r=n_c} f_r dr$:

$$\begin{aligned} A &\approx \frac{(m-1)}{1-n_c^{(1-m)}} && \text{if } m \neq 1, \\ A &\approx \frac{1}{\ln(n_c)} && \text{if } m = 1. \end{aligned}$$

For simplicity we will assume for $m \neq 1$ that n_c is large (meaning $\ln n_c \gg 1/|m-1|$). Then we have:

$$\begin{aligned} A &\approx \frac{1-m}{n_c^{(1-m)}} && \text{if } m < 1, \\ A &\approx m-1 && \text{if } m > 1. \end{aligned}$$

S1.3.2.2 Power-law frequency distributions capture a range of phenomenologies To illustrate the diverse range of phenomenologies encompassed by the power-law frequency distributions for different m, we will show how they differ in key characteristics.

The first characteristic we will compare is, how many NCs are larger or equal than the average frequency $\langle f_r \rangle = 1/n_c$. This is given by r_{ave}, the rank at which $f_{r_{\text{ave}}} = 1/n_c$.

The second characteristic we will compare is r_x, the number of largest NCs required to account for a fraction $0 < x \leq 1$ of all genotypes. This is approximated by taking the integral $x \approx \int_{r=1}^{r=r_x} f_r dr$ to give:

$$\begin{aligned} r_x &\approx \left(1 - x + x n_c^{-(m-1)}\right)^{-1/(m-1)} && \text{if } m \neq 1, \\ r_x &\approx n_c^x && \text{if } m = 1. \end{aligned}$$

These quantities highlight the differences between power laws with $m = 1$, $m > 1$, and $m < 1$ (in the limit of large GP maps with large L, n_p and n_c):

- For the pure Zipfian case $m = 1$:

$$r_{50\%} \approx n_c^{1/2}, \quad r_{\text{ave}} \approx \frac{n_c}{\ln(n_c)} \ll n_c, \quad \text{the smallest } f_{n_c} \approx \frac{1}{\ln(n_c) n_c} \ll \langle f_r \rangle = \frac{1}{n_c}.$$

  Only a small minority of the largest NCs account for half of all genotypes, and only a small minority of NCs are above-average in size.

- For the "super-Zipfian" case $m > 1$:

$$r_{50\%} \approx 2^{1/(m-1)}, \quad r_{\text{ave}} \approx (m-1)^{1/m} \cdot n_c^{1/m} \ll n_c, \quad f_{n_c} \approx \frac{m-1}{n_c^m} \ll \langle f_r \rangle$$

  To leading order in n_c, the number of large NCs accounting for half the genotypes, $r_{50\%}$, is independent of the number of NCs, and thus of the size of the GP map. Since the smallest NC must contain at least one genotype, this scaling is only valid for $(N_G/n_c^m) \geq 1/(m-1)$.

- For the "sub-Zipfian" case $0 < m < 1$:

$$r_{50\%} \approx 2^{-1/(1-m)} n_c \propto n_c, \quad r_{\text{ave}} \approx (1-m)^{1/m} n_c \propto n_c, \quad f_{n_c} \approx \frac{1-m}{n_c} \propto \langle f_r \rangle$$

  In contrast to the $m \geq 1$ case, for the sub-Zipfian case, both $r_{50\%}$ and r_{ave} are directly proportional to n_c and the smallest frequency is on the same order of magnitude as the average NC frequency.

These characteristics illustrate that the three options for the exponent m define a diverse set of GP maps. Thus, as a set they may cover key aspects of the spectrum of scaling behaviour for a wider range of GP maps, even though the exact frequency distributions of these GP maps are likely to be more complex than simple power-law forms.

S1.3.2.3 NC evolvability scaling To approximate the ruggedness, we also need to estimate the evolvability ϵ_r of the rth NC. Here, we make the ansatz that evolvability scales as a power law with frequency $\epsilon_r \propto f_r^{\beta}$, based on the relationships seen in our GP maps (with $0 \leq \beta \leq 1$, Fig. S4). To bound the normalisation constant, we note that the evolvability cannot exceed the total number of phenotypes n_p. Thus, we will write the highest evolvability corresponding to the largest NC (with rank $r = 1$) as $\epsilon_1 = \alpha n_p$, where

$\alpha \leq 1$ (for example, in one of the largest exhaustively studied GP maps, that of $L = 20$ RNA secondary structures, $\alpha > 0.75$ [16]).

Assuming that the power law scaling approximately holds for the full range of frequencies, without saturation effects at low or high frequencies (note that this is an approximation, for example in the Fibonacci model in Fig S4), we have the ansatz:

$$\epsilon_r = \alpha n_p \left(\frac{f_r}{f_1}\right)^{\beta} \tag{8}$$

In this scaling, the largest NC has an evolvability of $\epsilon_1 = \alpha n_p$, and the smallest NC (of rank n_c) an evolvability of $\epsilon_{\min} = \alpha n_p n_c^{-\beta m}$.

S1.3.2.4 Combining frequencies and evolvabilities to compute ruggedness Our scalings for the frequency distribution and evolvability, together with the fact that $f_r = |NC_r|/K^L$ by definition, allow us to write ruggedness as:

$$\begin{aligned}
\langle \text{fraction of genotypes in peaks} \rangle = R &= \sum_{r=1}^{n_c} \frac{f_r}{\epsilon_r + 1} \\
&\approx \frac{f_1^{\beta}}{\alpha n_p} \sum_{r=1}^{n_c} f_r^{1-\beta} \\
&\approx \frac{A}{\alpha n_p} \int_{r=1}^{r=n_c} \frac{1}{r^{m(1-\beta)}}\, dr \\
&\approx \frac{A}{1 - m(1-\beta)} \left[\frac{n_c^{m\beta}}{\alpha n_p} n_c^{(1-m)} - \frac{1}{\alpha n_p} \right] \qquad \text{if } m(1-\beta) \neq 1 \\
&\approx \frac{A}{(1 - m(1-\beta))\epsilon_{\min}} \left[n_c^{(1-m)} - n_c^{-\beta m} \right]
\end{aligned} \tag{9}$$

In the limit of large GP maps ($n_c \to \infty$) we find the following scaling:

- For $m = 1$, the expression in the square brackets is dominated by the first term, so the dominant scaling is $R \approx \frac{1}{\beta \ln n_c} \frac{1}{\epsilon_{\min}} \ll \frac{1}{\epsilon_{min}}$
- For $m > 1$, there are two cases:[4]
  First, $m - 1 < m\beta$, or equivalently $m < 1/(1-\beta)$ the dominant term is $\propto \frac{1}{n_c^{(m-1)} \epsilon_{\min}}$. If $m > 1/(1-\beta)$ (the large m limit) then ruggedness is dominated by the evolvability of the largest NC ($\epsilon_1 = \alpha n_p$) and $R \propto \frac{1}{n_c^{m\beta} \epsilon_{\min}}$. In either case, for large n_c, we have $R \ll \frac{1}{\epsilon_{min}}$.
- For $m < 1$ the dominant scaling is $R \approx \frac{1-m}{1-m(1-\beta)} \frac{1}{\epsilon_{\min}} \sim \mathcal{O}(\frac{1}{\epsilon_{\min}})$

To make further progress on our scaling arguments for the ruggedness, we need to make some assumptions about the minimum evolvability in the map, ϵ_{min}.

[4]Note that for $m > 1$, the power-law frequency distribution is only valid if the smallest NC contains at least one genotype, giving the condition $n_c^{m-1}/(m-1) \leq n_G/n_c$ (see section *Power-law frequency distributions capture a range of phenomenologies*). This limits m to a value that depends on the number of genotypes N_G and the number of NCs n_c.

S1.3.2.5 Scaling assuming the minimum evolvability $\epsilon_{\mathbf{min}} \approx \epsilon_{\mathbf{HoC}}$ Let us first start with a simple assumption about ϵ_{min}, namely that it is independent of the size of the smallest NC and that it scales like the evolvability of a single genotype in the HoC model, $\epsilon_{\text{min}} \approx \epsilon_{\text{HoC}} \propto 1/L$. On the one hand, this may be an underestimate of the true minimal evolvability since the smallest NC may have many genotypes, and so many more mutational neighbours than a single-genotype NC in the HoC model. On the other hand, the GP map evolvability needs to account for repeated phenotypes in a mutational neighbourhood, which reduce the evolvability compared to the HoC model: repeated phenotypes can occur by chance, depending on the phenotypic frequency distribution, or due to genetic correlations, i.e. the clustering of genotypes mapping to the same phenotype present in realistic GP maps [9].

With this assumption that $\epsilon_{\text{min}} \approx \epsilon_{\text{HoC}}$ (or higher ϵ_{min}), the ruggedness R of a GP map with $m \geq 1$ will be much smaller than $1/\epsilon_{\text{min}}$, and thus much smaller than that of the corresponding HoC baseline. This small ruggedness is due to the fact that larger, high-evolvability NCs take up such a large fraction of the genotype space for $m \geq 1$ that the smallest, low-evolvability NCs do not contribute to the dominant scaling in the ruggedness calculations.

For $m < 1$, on the other hand, the assumption that $\epsilon_{\text{min}} \approx \epsilon_{\text{HoC}}$ leads to a ruggedness that scales like the ruggedness in the HoC model. However, for $m < 1$ the assumption that $\epsilon_{\text{min}} \approx \epsilon_{\text{HoC}}$ is most likely to be an underestimate since even the smallest NC is relatively large, on the same order of magnitude as the mean NC size (see section *Power-law frequency distributions capture a range of phenomenologies*) .

S1.3.2.6 Scaling when the minimum evolvability $\epsilon_{\mathbf{min}}$ grows with the size of the smallest NC Let us revisit the $m < 1$ case, where even the smallest NC is on the same order of magnitude as the mean NC size (see section *Power-law frequency distributions capture a range of phenomenologies*), and thus likely to have a higher evolvability than single-genotype NCs in the HoC model. For example, we might assume that a NC made up of x genotypes should have access to a x-times as many distinct phenotypes than a NC made up of a single genotype in the HoC model. This would be an overestimate because it ignores repeated phenotypes, either due to diminishing returns (if the same high-frequency phenotypes appear in the neighbourhood repeatedly due to phenotypic bias [17]) or due to genetic correlations [9]. Thus, the scaling might be non-linear, as in our initial evolvability scaling, where frequency differences *within* a GP map are related to evolvability differences *within* a GP map via a power law with exponent $\beta < 1$ (see eq. (8)). However, we do not have to assume a specific functional form: we simply assume that $\epsilon_{min} \gg \epsilon_{HoC}$ as long as the minimum NC size is much larger than one, i.e. $K^L f_{n_c} \gg 1$. With this assumption, the ruggedness, which scales as $\epsilon_{\text{min}}^{-1}$ for the $m < 1$ case, will be much lower than the HoC ruggedness, i.e. $R \ll R_{\text{HoC}}$.

To sum, up, we get the following intuitive result: if even the smallest NCs are large and have much higher evolvabilities than a single-genotype NC in the HoC model, then the ruggedness is much smaller than in the HoC expectation even for the $m < 1$ power laws.

S1.3.2.7 Scaling for peak height distribution Having discussed the implications of our f_r- and ϵ_r-scalings for ruggedness, we next turn to our approximation for the peak height distribution.

$$P_{\text{peaks}}(F \leq G) \approx \frac{1}{\sum_{\text{NC index i}} (\epsilon_i + 1)^{-1}} \sum_{\text{NC index i}} \frac{C_F(G)^{\epsilon_i + 1}}{\epsilon_i + 1} \tag{10}$$

Since $C_F(G)$ is a CDF with $C_F(G) \leq 1$, we can put the following upper bound on this expression:

$$P_{\text{peaks, GP map}}(F \leq G) \lessapprox C_F(G)^{\epsilon_{min}+1} \frac{1}{\sum_{\text{NC index i}}(\epsilon_i + 1)^{-1}} \sum_{\text{NC index i}} \frac{1}{\epsilon_i + 1}$$
$$\lessapprox C_F(G)^{\epsilon_{min}+1} \quad (11)$$

Since all our GP maps under any scaling exponent contain NCs with $\epsilon_r > \epsilon_{min}$, the equality only holds if $C_F(G) = 1$, i.e. when exceeding the maximum fitness value in the PF map, such that all genotypes and thus all peaks have fitness values below G. The same logic can be applied to obtain a lower bound:

$$P_{\text{peaks, GP map}}(F \leq G) \gtrapprox C_F(G)^{\epsilon_{max}+1} \frac{1}{\sum_{\text{NC index i}}(\epsilon_i + 1)^{-1}} \sum_{\text{NC index i}} \frac{1}{\epsilon_i + 1}$$
$$\gtrapprox C_F(G)^{\epsilon_{max}+1}$$

Thus, we have:

$$C_F(G)^{\epsilon_{max}+1} \lessapprox P_{\text{peaks, GP map}}(F \leq G) \lessapprox C_F(G)^{\epsilon_{min}+1} \quad (12)$$

Note that these calculations started with an approximate peak height distribution (eq. 10), and thus $\lessapprox$ signs are used.

First, let us compare to the HoC landscape, where each single-genotype NC has an evolvability ϵ_{HoC}, giving $P_{\text{peaks, HoC}}(F \leq G) \approx C_F(G)^{\epsilon_{HoC}+1}$ (see ref [18]). As long as $\epsilon_{min} \geq \epsilon_{HoC}$, we have $P_{\text{peaks, HoC}}(F \leq G) \gtrapprox P_{\text{peaks, GP map}}(F \leq G)$. Thus, our approximate calculations suggest that the expected fraction of peaks that are of low fitness, $P_{\text{peaks}}(F \leq G)$, is lower in the GP-map-based landscape than in the corresponding HoC model, as long as $\epsilon_{min} \geq \epsilon_{HoC}$ (for more context on this assumption, see the previous section).
Next, let us consider eq. (12) for the specific case of a uniform distribution underlying the PF map, with $C_F(G) = F$ for $0 \leq F \leq 1$. Then, as long as $\epsilon_{min} \gg 1$ for large maps, the upper bound gives $P_{\text{peaks}}(F \leq G) \ll 1$ for $G < 1 - \delta$ and a small δ. For $G = 1$, we will have $P_{\text{peaks}}(F \leq 1) = 1$. Thus, most peaks are expected to have a fitness close to $F = 1$. Since this argument applies whenever $\epsilon_{NC} \gg 1$, it applies to both the HoC and the GP-map-based landscape, in agreement with previous results on peak heights in the HoC model [18]. This argument is consistent with our finding that the peak-evolvability-height relationship saturates for uniform PF maps (see Fig. S11). Thus, even though we expect differences in the peak height distribution if $\epsilon_{min} \geq \epsilon_{HoC}$, the effect size can be small.

S1.3.2.8 Discussion: testing extrapolations through sampling The big question then is whether these extrapolations will hold for realistic, biophysical GP maps in the long-L limit. Here some caution is needed. While the power-law assumption permits a broad range of qualitatively different frequency distributions (see section *Power-law frequency distributions capture a range of phenomenologies*), it needs to be tested against GP maps in the large-L limit. For this, we cannot simply rely on scaling laws for phenotype frequencies since the genotypes mapping to a single phenotype (a *neutral set*) can fragment into disconnected neutral components for a variety of reasons, including biophysical reasons such as base pairing in the RNA secondary structure GP map [19]. Similarly, our power-law assumption for the scaling of evolvabilities with frequencies, as well as our assumptions about minimum evolvabilities, need to be tested in large-L GP maps. Since exhaustive analysis will not be feasible for large-L GP maps, frequencies and evolvabilities will have

to be estimated using sampling methods. Such analyses have successfully revealed the scaling of *phenotypic* quantities for RNA secondary structures [15] (such as number of phenotypes in the map) and a sample-based method for NC *sizes* exists [20]. However, more work is needed to develop accurate sampling approaches to estimate NC *evolvabilities* [21].

S2 Supplementary computational analyses

S2.1 Additional NC graph analyses

S2.1.1 Number, evolvabilities and sizes of NCs

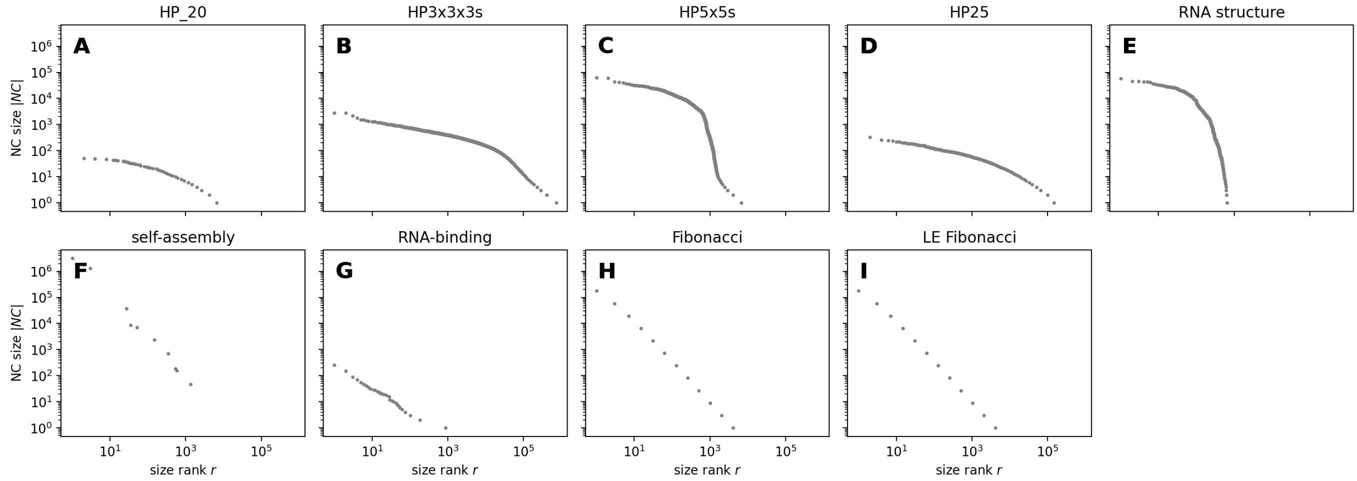

Figure S3: **Distribution of NC sizes $|NC|$:** in the scaling calculations (section S1.3), the distribution of NC sizes is important. Thus, for each GP map, we take each NC and plot its NC size $|NC|$ against the NC size rank, i.e. the number of NCs with equal or higher size.

In our ruggedness predictions, the number, evolvabilities, and sizes of NCs are crucial. In Table 1, we show the number of NCs and further key quantities for each GP map from the main text. This also includes the prevalence of the deleterious phenotype, which represents a single qualitatively distinct phenotype in each map, for example, non-terminating and non-deterministic assemblies in the self-assembly model. We also show the size distribution of NCs in Fig. S3, and the relationship between NC size and NC evolvability in Fig. S4.

As expected from prior literature, see e.g. [19, 29], we find that large NCs tend to be more evolvable in all GP maps except in the low-evolvability Fibonacci model, where evolvability is low for all phenotypes by construction and decreases with NC size [6]. We further note that, out of all biophysical GP maps, the self-assembly GP map has the flattest slope in the logarithmic NC-size-evolvability plot, indicating that even large NCs only reach moderate evolvabilities. Thus, even large NCs have a comparatively high likelihood of being peaks and the ruggedness (Fig 2 in the main text) is comparatively high in this map. However, this feature may be an artefact of the limited size of the model in particular [1]: with a sequence length of $L = 8$, only 13 distinct viable phenotypes exist, and therefore evolvabilities have to be integers between $\epsilon_{NC} = 0$

GP map	sequence length L	alphabet size K	number NCs	number genotypes $N_G = K^L$	number phenotypes n_p	number zero-evolv. NCs	mean size zero-evolv. NCs	prevalence deleterious phenotype
HP_20	20	2	6,586	1,048,576	5,310	2,067	2	98 %
HP3x3x3s	27	2	732,157	134,217,728	49,807	151,640	1	94 %
HP5x5s	25	2	6,785	33,554,432	549	586	1	82 %
HP25	25	2	148,254	33,554,432	107,336	36,872	2	98 %
RNA structure	12	4	645	16,777,216	57	0		85 %
self-assembly	8	8	1,347	16,777,216	13	0		54 %
RNA-binding	7	4	874	16,384	313	5	1	85 %
Fibonacci	12	3	4,095	531,441	4,095	0		1 %
LE Fibonacci	12	3	4,095	531,441	4,095	1	177,147	1 %

Table 1: **Characteristics of the GP map models used throughout this paper:** for each GP map, the table gives the sequence length L, alphabet size K, number of NCs, the number of genotypes $N_G = K^L$ (which includes those folding to the deleterious phenotype), the number of distinct viable phenotypes, the number of isolated NCs with $\epsilon_{NC} = 0$, the mean size of isolated NCs and the prevalence of the deleterious phenotype among all genotypes. The biophysical GP maps are constructed from Greenbury et al.'s [1] datasets (several characteristics are duplicated from a table in ref [1] for completeness, but without counting the deleterious phenotype among the phenotypes), and are based on the following models: the HP lattice models of protein tertiary structure [22, 23], the Polyomino self-assembly tile model of protein quaternary structure (proposed in this form in [24], but based on a longer history of tile-assembly models, see [25]) and a free-energy-based RNA secondary structure folding model implemented in the ViennaRNA package [26]. The data underlying the RNA-binding map was curated by Payne and Wagner [27] from the CISBP-RNA database, and we constructed a categorical, many-to-one GP map from this data as described in the text. Finally, we have the Fibonacci model and its low-evolvability version (adapted from the "gene-like model" and the corresponding reference model [6], which is a generalisation of the original Fibonacci model [28]), with a minor adjustment to guarantee symmetry of allowed mutations as described in the main text.

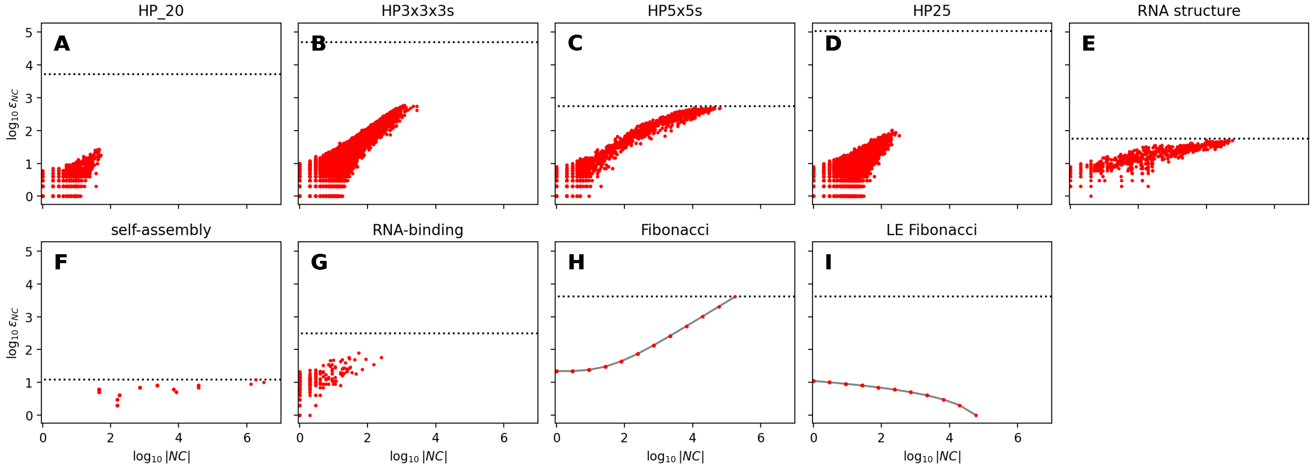

Figure S4: **Large NCs with high $|NC|$ tend to be more evolvable (higher ϵ_{NC}), except in the low-evolvability Fibonacci map.** The relationship between NC size and evolvability is important for the prevalence of peaks in genotype space. Thus, for each GP map, we take each NC and plot the logarithm of the NC evolvability $\log_{10} \epsilon_{NC}$ against the logarithm of the NC size $\log_{10} |NC|$ (excluding zero-evolvability NCs values due to the log scale). In addition to the computational data (red), analytic relationships are shown for the Fibonacci models (grey, eqs (4-5)). The maximum possible evolvability $n_p - 1$ (where n_p is the number of phenotypes in the map) is drawn as a black dotted line.

and $\epsilon_{NC} = 12$. The number of phenotypes is higher for longer sequences and larger alphabets [24], but these GP maps are difficult to store and analyse exhaustively because the number of genotypes grows rapidly with sequence length as K^L, especially for the large alphabets typical of the self-assembly models.

Moreover, we note that some GP maps have completely isolated NCs with $\epsilon_{NC} = 0$ (i.e. NCs with mutational connections only to the deleterious phenotype). These isolated NCs are particularly common in the HP protein model and constitute one of the differences between this model and the RNA secondary structure model (see [30, 31] for a more detailed discussion on qualitative and quantitative differences). Such isolated NCs are guaranteed to be peaks under any fitness assignment. Since it is unclear how such isolated peaks would be reached by evolving populations, we repeat our ruggedness analysis with isolated peaks excluded in section S2.2.2.

S2.1.2 Treatment of evolvabilities for main text Fig.6

In Fig. 6 in the main text, all navigability simulations were based on the full NC graph, but to obtain a single evolvability value representing each NC graph, we took the geometric mean of all NC with zero-evolvability NCs treated as $\epsilon_{NC} = 0.01$. Here, we compare several alternatives: one could take the geometric mean, arithmetic mean or the median of all evolvabilities. Fig. S5 illustrates that the order of magnitude of the typical evolvability, which is key in our navigability analysis, does not depend strongly on this choice.

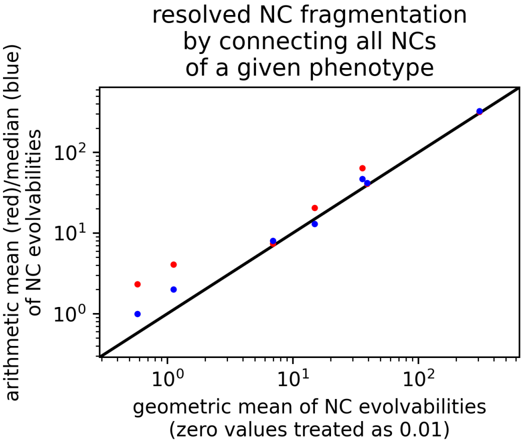

Figure S5: **Comparisons of the median and arithmetic/geometric mean evolvability in a NC graph:** In Fig. 6 in the main text, we use the geometric mean to get a representative evolvability value for a NC graph (with zero values treated as 0.01, such that a single zero-evolvability NC does not set the mean to zero). Here, we compare this geometric mean (x-axis) to the arithmetic mean (red) and the median (blue) for the GP maps included in Fig. 6.

S2.1.3 NC graph network analysis

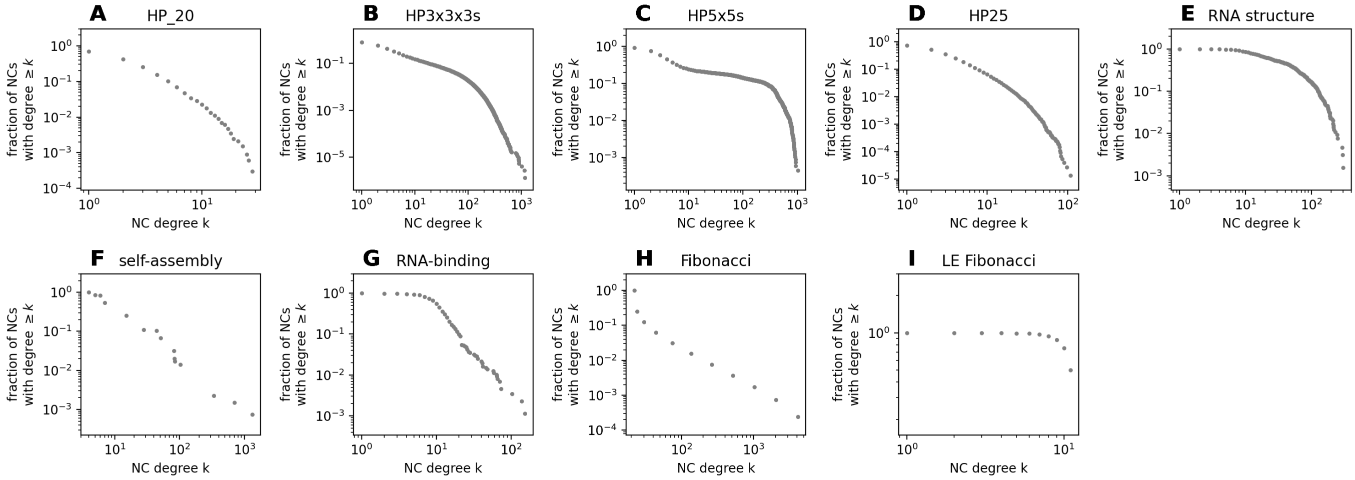

Figure S6: **Degree distribution of NC graphs:** For each NC graph, we plot the cumulative degree distribution, i.e. the fraction of nodes with degree k or greater as a function of the degree k. In all networks, except the low-evolvability Fibonacci model, the degrees span several orders of magnitude.

Since NC graphs are at the centre of our analysis, we next turn to their network properties. First, we focus on the degree distribution of our NC graphs (Fig. S6). The network degree of a NC in its NC graph is closely related to its NC evolvability, but the two are not identical: for example, a NC of interest can

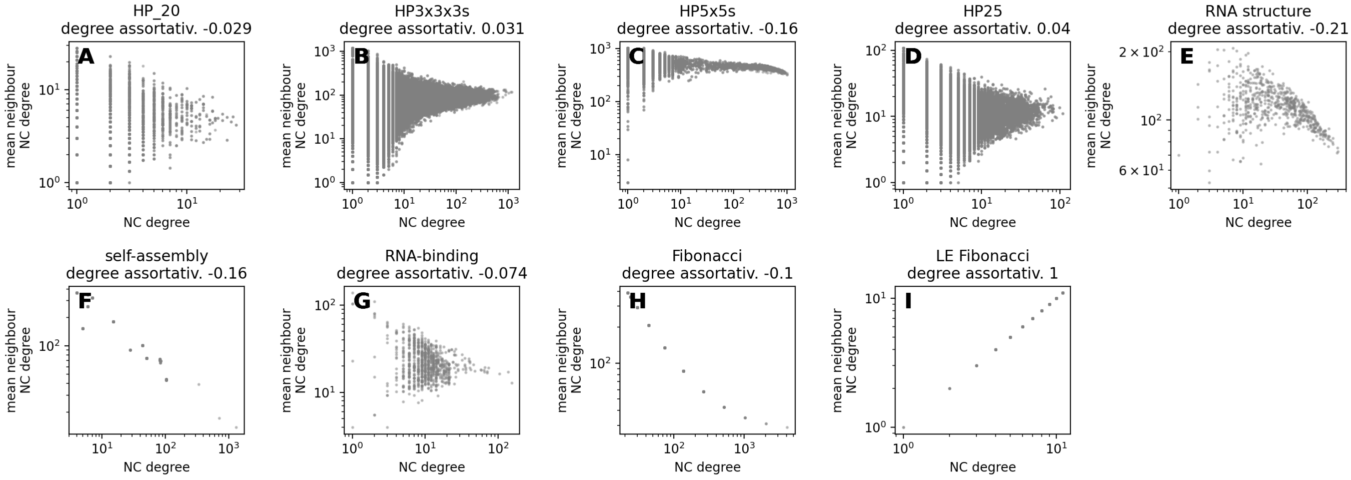

Figure S7: **Degree assortativity in NC graphs:** we take each node in a NC graph and plot the mean degree of its neighbouring nodes against its own degree. We summarise this data by computing the degree assortativity coefficient with NetworkX [32] (see plot titles). NC graphs tend to have low or negative assortativity (again the low-evolvability Fibonacci model is an outlier by definition).

be connected to two NCs of phenotype p_x, and this would contribute two units to its degree in the NC graph, but only one unit to its evolvability. We find that the NC graph degrees vary over several orders of magnitude (Fig. S6). This is not surprising given that evolvability and thus degree is correlated with NC size (see Figs. S3-S4). Some of these networks are approximately scale-free, i.e. their cumulative degree distributions in Fig. S6 follow a power law (see ref [33] for a more detailed definition).

Having quantified *how many* NCs each NC is connected to, let us focus on *which* NCs it tends to be connected to. In particular, we analyse whether high-degree nodes tend to be connected to other high-degree nodes (Fig. S7), i.e. whether the network has degree assortativity. We find mixed results: our NC graphs have negative or small positive degree assortativity (again with the LE Fibonacci model as an outlier). Our finding does not invalidate previous observations of a positive degree assortativity in GP maps [34] since these previous results focused on a different scale: the network of genotypes in a NC, rather than the networks of NCs in the full GP map.

S2.2 Additional ruggedness analyses

In this section, we will provide additional data related to the ruggedness analysis in Fig. 3 of the main text.

S2.2.1 Changes to the underlying GP maps, including dimensionality and correlations

One key takeaway from the calculations in the main text is that the mean ruggedness depends only on the set of all NC sizes $|NC|$ and evolvabilities ϵ_{NC}. Other GP map features only play an indirect role through their correlation with these properties. In this section, we test this indirect dependency by revisiting an analysis from Greenbury et al. [1] and varying two GP map characteristics: (1) The *dimensionality*, i.e. the number of positions allowed to mutate per genotype, and (2) *genetic correlations*, i.e. the clustering of genotypes of

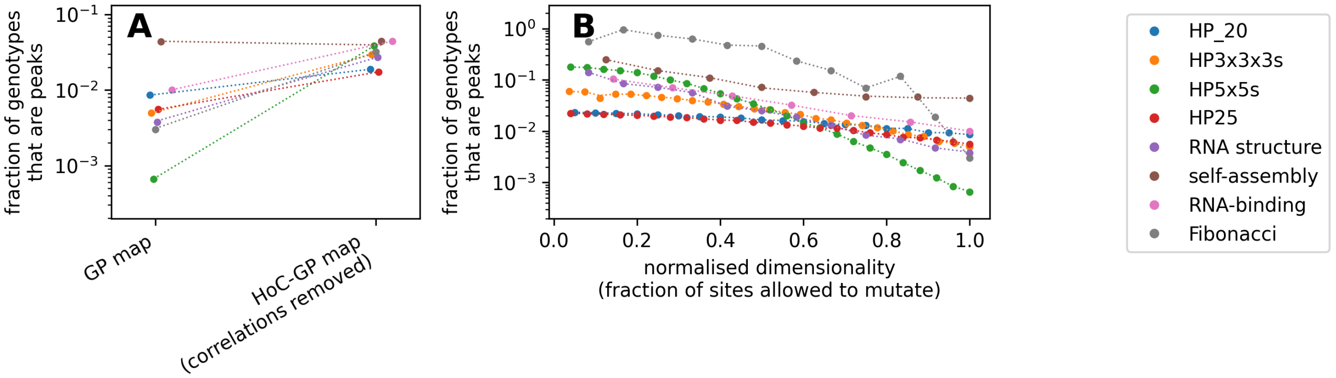

Figure S8: **Perturbing the correlations and dimensionality in a GP map indirectly affects ruggedness by altering the numbers, sizes and evolvabilities of NCs:** (A) We compare the ruggedness in each GP map to that of a corresponding zero-correlation GP-HoC map (the "random GP map" in [9]). (B) We compute the ruggedness in reduced-dimensionality GP maps, where only a fraction of all L sites are allowed to mutate (see ref [1]). In both plots, scatter points denote the mean ruggedness of each map in simulations. Dotted lines illustrate the prediction based on NC sizes and evolvabilities: $\langle \text{ruggedness} \rangle = K^{-L} \sum_{\text{NC indices } i} |NC_i| (\epsilon_i + 1)^{-1}$. We find that changes in the set of NC sizes and evolvabilities fully account for changes in ruggedness. Each data point is based on a single GP map (for example, a single HoC-GP map, or a reduced-dimensionality GP map with a fixed set of mutating sites). Computational ruggedness estimates are based on 10^3 PF maps for each GP map. Slight x-axis offsets are used in (A) to show overlapping points more clearly. The LE Fibonacci model is not shown because its construction already relies on excluding certain mutations. The data from the main text, i.e. for unmodified GP maps, is shown as "GP map" in part (A) and as "normalised dimensionality = 1" in part (B).

the same phenotype [9]. This clustering means that two mutationally connected genotypes are more likely to map to the same phenotype than in the corresponding uncorrelated null model. Thus, genetic correlations are closely linked to the high prevalence of neutral mutations in GP maps, and the formation of large NCs [9].

To investigate the role of these two GP map features, we follow Greenbury et al. [1, 9] and perturb them in two ways: (1) The dimensionality can simply be reduced by limiting the number of mutating sites in a genotype [1]. (2) The genetic correlations can be removed by building an uncorrelated *GP-HoC model* for a given GP map [9], by shuffling the assignment of phenotypes over genotypes. This was originally introduced as the "random GP map", but here we use the term GP-HoC model to highlight parallels with uncorrelated House-of-Card models.

When we reduce the dimensionality and genetic correlations of our GP maps, we find that their ruggedness tends to increase (Fig. S8), as expected. These results, as well as the fact that the self-assembly model forms an exception due to its low number of phenotypes (see section S2.1.1), are consistent with Greenbury et al.'s [1] ruggedness/navigability analyses for lower-dimensionality and uncorrelated maps.

Having shown the computational ruggedness results, we turn to our ruggedness predictions (dotted lines in Fig. S8), which are based only on the set of NC sizes $|NC|$ and NC evolvabilities ϵ_{NC}. These predictions continue to be accurate, indicating that the changes in NC sizes and evolvabilities fully predict the changes in mean ruggedness. These changes in NC sizes $|NC|$ and NC evolvabilities ϵ_{NC} can arise from

GP map perturbations in many ways: For example, reducing dimensionality implies reducing the number of allowed mutations, which can lower NC evolvabilities by removing mutations connecting two NCs. Alternatively, reducing the number of allowed mutations can fragment an initially connected NC into several lower-evolvability NCs. Similarly, the reduction of genetic correlations through randomisation can break NCs into several lower-evolvability NCs. Thus, concepts like dimensionality and genetic correlations are indeed important for the ruggedness in GPF maps, but this effect is an indirect one mediated by their effects on the numbers, sizes and evolvabilities of NCs.

S2.2.2 The impact of deleterious phenotypes and zero-evolvability NCs on ruggedness

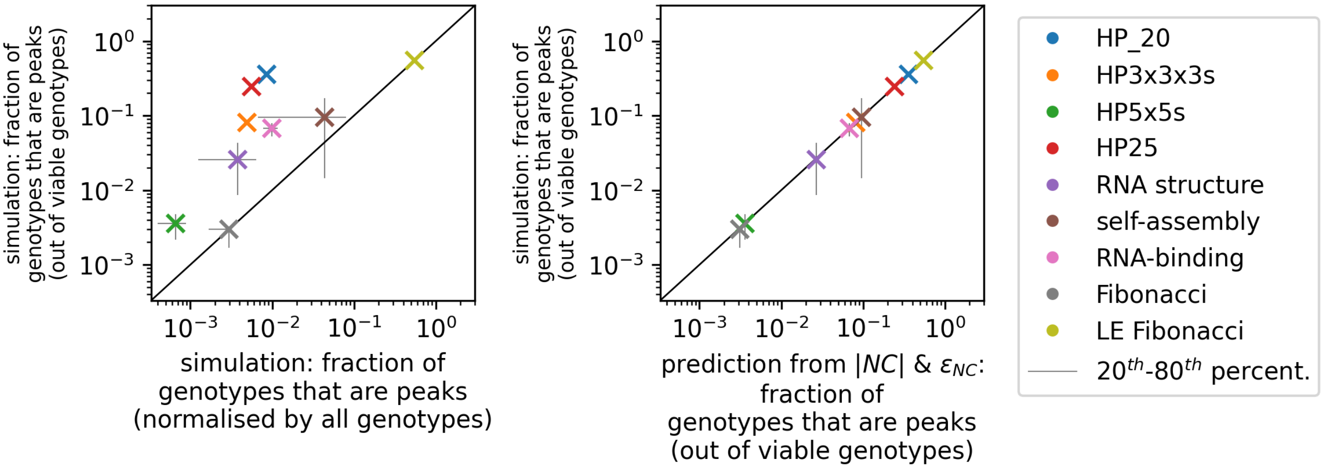

Figure S9: **Prevalence of peaks normalised relative to the set of all viable genotypes (i.e. genotypes not mapping to the deleterious phenotype):** (A) Simulation data comparing the renormalised fraction of peaks (y-axis) to the fraction relative to all genotypes (x-axis, this is the data from the main text). (B) The mean renormalised fraction of genotypes in peaks for part A (y-axis) is well-predicted by NC sizes $|NC|$ and NC evolvabilities ϵ_{NC} using eq. (13) (x-axis).

Finally, we analyse the impact of two GP map peculiarities on ruggedness estimates: the presence of deleterious phenotypes and the existence of zero-evolvability NCs.

Qualitatively distinct phenotypes exist in all our GP maps: For example, non-uniquely folding sequences in the HP model, non-deterministic or non-finite outcomes in the self-assembly model and structures without base pairs in the RNA secondary structure map [1] (however, for the case of RNA, they are much less prevalent for longer, more biologically relevant sequence lengths [15]). Similarly, some sequences in the RNA-binding model do not bind a single RNABP, and genotypes in the Fibonacci model are considered undefined if they lack a stop codon [6]. Throughout this paper, we follow the worst-case treatment of Greenbury et al. [1], and treat all genotypes with these criteria as deleterious and of zero fitness. Thus, they cannot form accessible paths or escape routes from peaks and are excluded from the NC graph.

Since genotypes mapping to the deleterious phenotype cannot form peaks, it may be useful to exclude them from the ruggedness normalisation and instead normalise the ruggedness of our GP maps relative to the number of viable genotypes $\sum_{\text{NC indices } i} |NC_i|$ as follows (i.e. genotypes mapping to any phenotype except

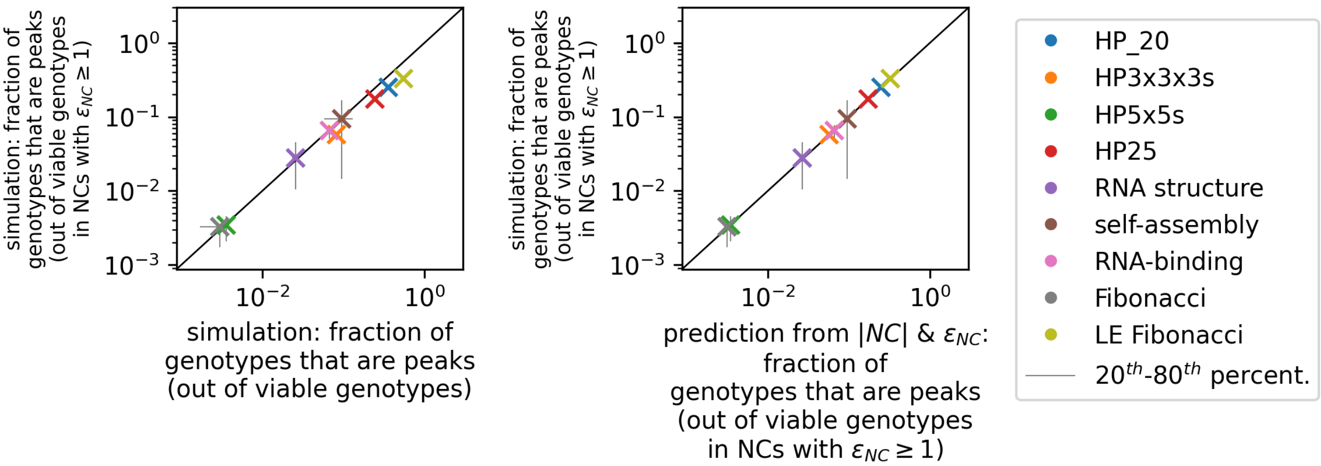

Figure S10: **Prevalence of peaks relative to the number of genotypes in viable & non-isolated NCs (with $\epsilon_{NC} > 0$):** (A) Simulation data comparing the renormalised fraction of peaks among viable, non-isolated NCs (y-axis) to the fraction relative to all viable NCs (x-axis, this is the data from Fig. S9). (B) The mean renormalised fraction of genotypes in peaks from part A (y-axis) is well-predicted by NC sizes $|NC|$ and NC evolvabilities ϵ_{NC} using eq. (14) (x-axis).

the deleterious phenotype):

$$\langle \text{ruggedness} \rangle = \frac{1}{\sum_{\text{NC indices } i} |NC_i|} \sum_{\text{NC indices } i} \frac{|NC_i|}{\epsilon_i + 1} \tag{13}$$

Fig. S9 shows that the renormalised mean ruggedness is well-predicted by eq. (13).

Next, we turn to zero-evolvability NCs, which only have the deleterious phenotype in their mutational neighbourhood. They technically form a peak under any PF map as long as the deleterious phenotype is treated as zero-fitness. However, such peaks may not be relevant for evolutionary processes, since such mutationally isolated NCs cannot be reached by mutations, and are unlikely to arise as initial conditions. We therefore further normalise our predicted and simulated number of peaks relative to all viable genotypes that are not part of zero-evolvability NCs (Fig S10):

$$\langle \text{ruggedness} \rangle = \left(\sum_{\text{NCs indices with } \epsilon_i > 0} |NC_i| \right)^{-1} \times \sum_{\text{NCs indices with } \epsilon_i > 0} \frac{|NC_i|}{\epsilon_i + 1} \tag{14}$$

In Fig. S10, we see that additionally excluding zero-evolvability NCs has a minor impact on our results because zero-evolvability NCs tend to be small with low $|NC_i|$ (see table. 1), except for the single zero-evolvability NC in the low-evolvability Fibonacci model. Moreover, the renormalised mean ruggedness continues to be well-predicted by NC evolvabilities and sizes (eq. (14)).

We note that drawing the line between viable and deleterious may be more difficult in more complex biological GP maps. The point here is that our formalism can still take unviable mutations into account.

S2.3 Additional analyses on the expected height of a given peak of evolvability ϵ

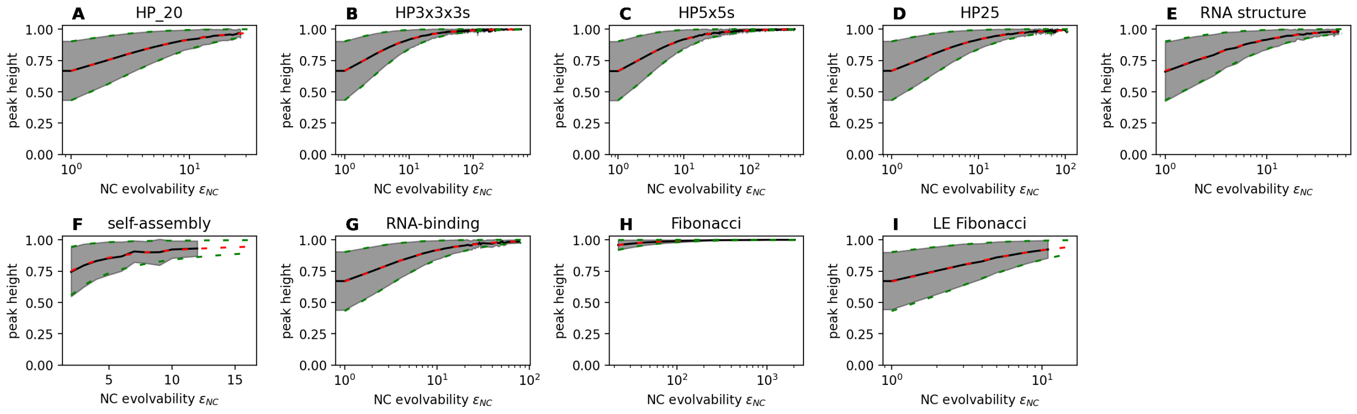

Figure S11: **High-evolvability peaks have high expected fitness:** each subplot shows data for one GP map. We simulate GPF maps sampled from a uniform distribution between 0 and 1, and plot peak fitness against evolvability (mean in grey and standard deviation as shaded grey area). In agreement with the analytic predictions from eq. (15) (mean and standard deviation in red and green), high-evolvability peaks tend to be high in fitness. The simulation results are based on 10^3 PF map realisations for each GP map, with up to 5×10^6 peaks per GP map included in the plot. The RNA structure GP map data is duplicated from the main text for completeness.

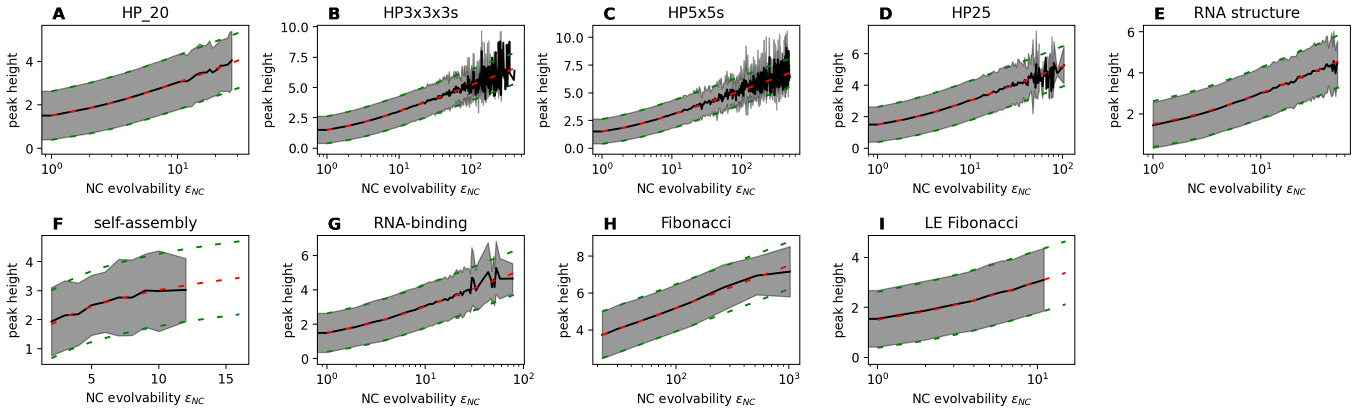

Figure S12: **Same as Fig. S11, but for a PF map sampled from an exponential distribution with rate parameter $\lambda = 1$.**

In the main text, we found analytic expressions for the expected fitness of a peak NC as a function of its NC evolvability ϵ_{NC}. We found that high-evolvability NCs tend to form high-fitness peaks, if they are peaks at all. Specifically, for PF maps drawn from a uniform distribution between 0 and 1, we have:

$$\langle F \rangle_{\text{peak}} = \frac{\epsilon_{NC} + 1}{\epsilon_{NC} + 2}; \quad \text{Var}(F_{\text{peak}}) = \frac{\epsilon_{NC} + 1}{(\epsilon_{NC} + 3)(\epsilon_{NC} + 2)^2} \tag{15}$$

And for PF maps drawn from an exponential distribution with $\lambda = 1$, we have:

$$\langle F \rangle_{\text{peak}} = \sum_{k=1}^{\epsilon_{NC}+1} \frac{1}{k} \qquad \text{Var}(F_{\text{peak}}) = \sum_{k=1}^{\epsilon_{NC}+1} \frac{1}{k^2} \tag{16}$$

This relationship was applied only to the RNA structure map in the main text, and so data for all GP maps is shown in Figs S11 & S12. We find that the analytic predictions are in excellent agreement with simulation data. Interestingly, the Fibonacci map has no low-evolvability NCs with $\epsilon < 22$, leading to a weaker trend in the saturating evolvability-height relationship of eq. (15) for a uniform map. However, for the exponential distribution (Fig. S12), which does not have a fixed upper limit, mean fitness continues to increase with NC evolvability, without the saturation seen for the uniform distribution, approximately as $\int_1^{\epsilon_{NC}+1} x^{-1} dx = \log(\epsilon_{NC} + 1)$.

The link between neighbourhood sizes (here evolvabilities) and peak heights holds beyond GPF maps: for example, in the HoC model, peaks tend to be higher for landscapes with larger mutational neighbourhoods [18]. However, there is one key difference: In the HoC model, the neighbourhood size is typically set by the sequence length L and alphabet size K, and thus takes a single value for an entire landscape. In GP maps, however, the relevant neighbourhood size is the evolvability, and this can differ by orders of magnitude *within* a single landscape and is correlated with further features like NC sizes, creating a rich phenomenology with peaks of different heights, evolvabilities and sizes.

The relationship between peak evolvability and height also implies an indirect link between peak size $|NC|$ and height: larger NCs tend to have higher evolvability (see Fig S4) and thus, if they form peaks, they tend to form higher peaks. This positive trend could be weak, because it is an indirect effect and because neither the link between NC size and evolvability nor the link between evolvability and peak height is one-to-one (for the latter, the mean and standard deviation are given by eqs (15&16)). This argument is consistent with the (weakly) positive trends seen in Figs S13 & S14 and with the fact that the lowest peaks tend to correspond to smaller NCs compared to the mean peak size in the landscape (Figs S15 & S16). The trend is missing in the self-assembly GP map, consistent with the fact that even large NCs in this map only reach moderate evolvabilities (see Fig S4), and the trend is not very clear in the RNA-binding map, where the log-size-evolvability data shows a lot of scatter around the central trend (see Fig S4). In the low-evolvability Fibonacci model, the trend is reversed, precisely because the link between peak size and height is an indirect one: larger peaks are of higher fitness only if NC evolvability is *positively* correlated with NC size, which is not the case in the low-evolvability Fibonacci model (see Fig. S4). While the low-evolvability Fibonacci model is useful for elucidating this relationship and disentangling direct and indirect links, it is not a realistic GP map.

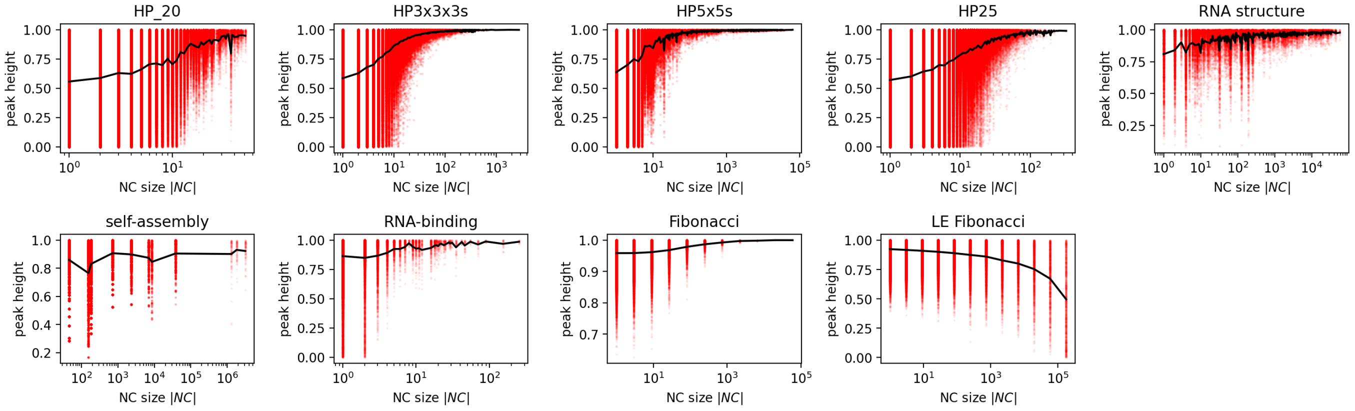

Figure S13: **In GP maps, where NCs with larger NC size are more evolvable, we expect a (weakly) positive trend between peak size and peak height/fitness:** here the peak heights from Fig. S11 (for PF maps drawn from a uniform distribution between 0 and 1) are plotted against NC size rather than NC evolvability. Red scatter points represent individual peak NCs. The average fitness for each fixed NC size in the data is shown as a black line. The negative trend in the low-evolvability Fibonacci model is expected since more evolvable NCs are smaller in this model.

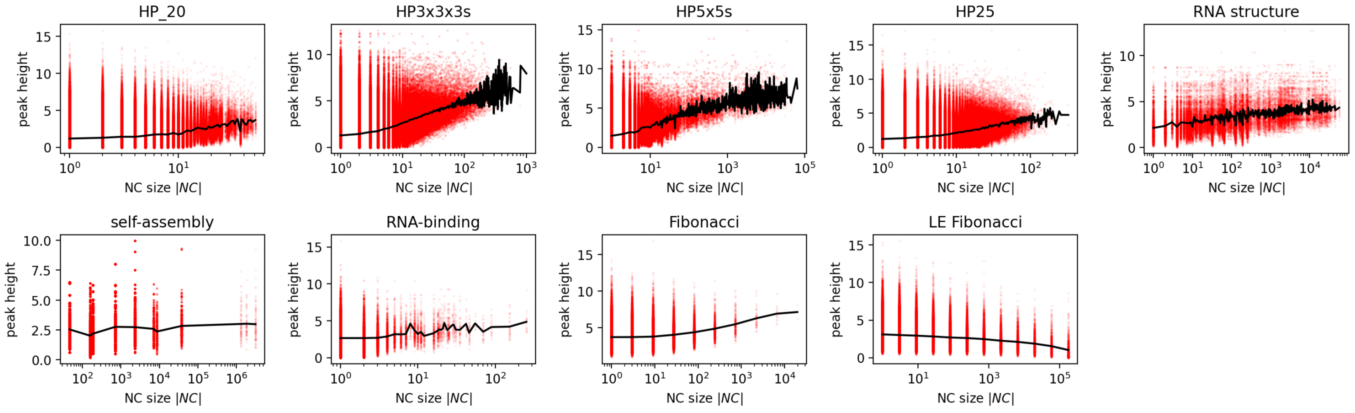

Figure S14: **Same as Fig. S13, but for a PF map drawn from an exponential distribution with rate parameter $\lambda = 1$.**

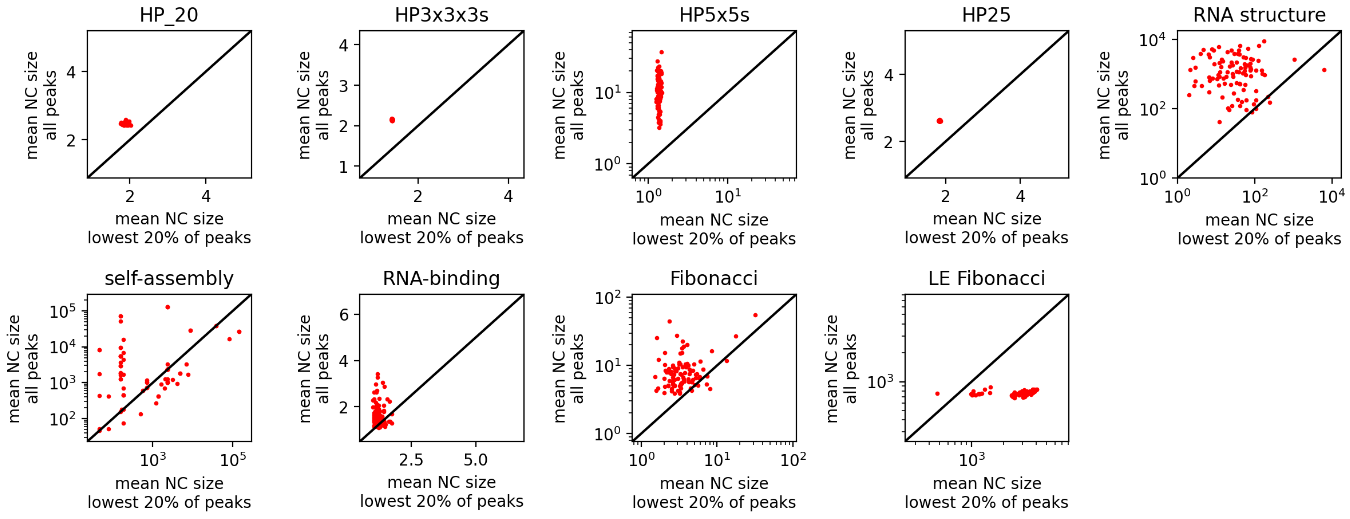

Figure S15: **Do the lowest peaks correspond to small NCs?** For the first 100 GPF maps in our peak data (uniform distribution), we plot the mean NC size of all peaks in each GPF map against the mean NC size of the lowest-20% of peaks in the same GPF map. We find that the lowest peaks tend to have lower NC sizes, except for the self-assembly GP map, where the trend is unclear. The trend is reversed in the low-evolvability Fibonacci model, which has a negative NC-size-evolvability relationship. Only GPF maps with over 20 peaks are included in the statistics.

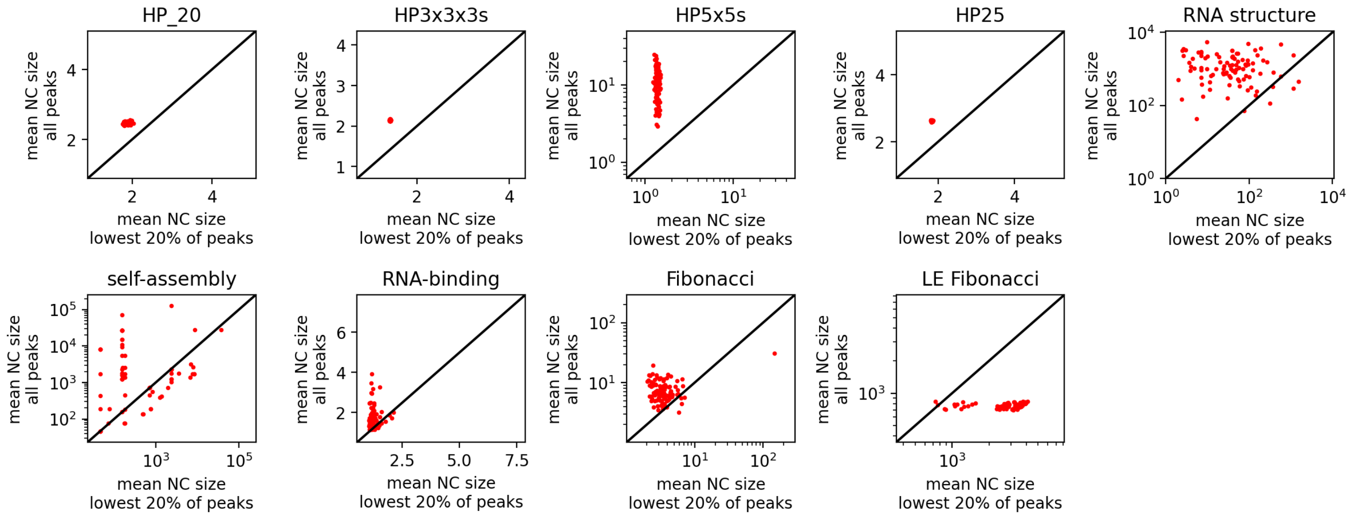

Figure S16: **Same as Fig. S15, but for PF maps drawn from an exponential distribution with rate parameter $\lambda = 1$.**

S2.4 Additional analyses on peak height distributions

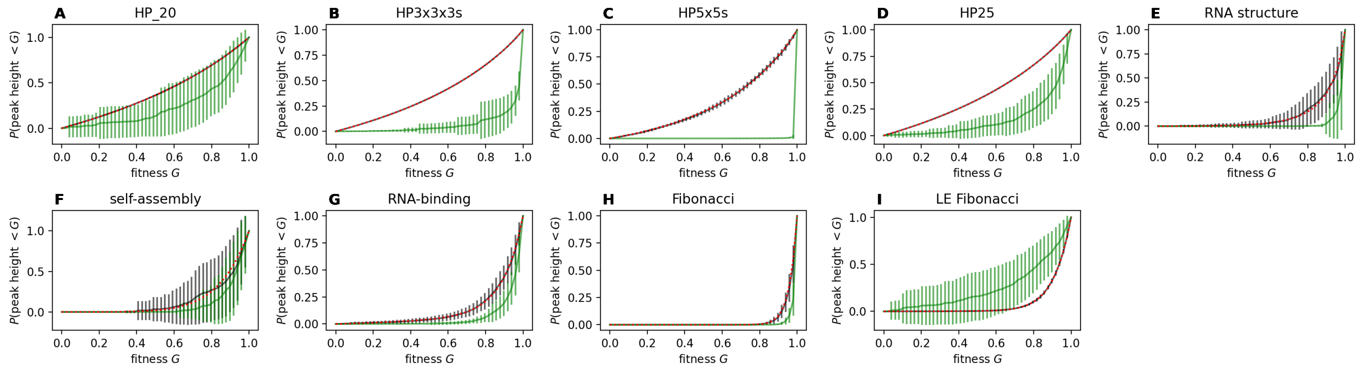

Figure S17: **Peak height distribution when the PF map is based on a uniform distribution:** each subplot focuses on one GP map - see subplot titles. For each GP map, we take the first 100 PF maps (uniform distribution between 0 and 1). Then, we compute the fraction of all peaks in each resulting landscape with fitness $F < G$ (black, mean across all 100 landscapes is shown, with standard deviation as error bars). This distribution is well-approximated by eq. (17) (red). Turning to the peaks reached by an adaptive walk starting from a genotype that is lower in fitness than the lowest-fitness peak (green, again mean and standard deviation across GPF maps), we find that populations tend to preferentially reach high peaks, except in the low-evolvability Fibonacci model. The lowest-fitness phenotype can form peaks if it has zero-evolvability NCs, and in this case, it is not possible to start adaptive walks from a lower-fitness genotype than the lowest peak. Such GPF maps are therefore excluded from the analysis. The RNA structure GP map data is duplicated from the main text for completeness.

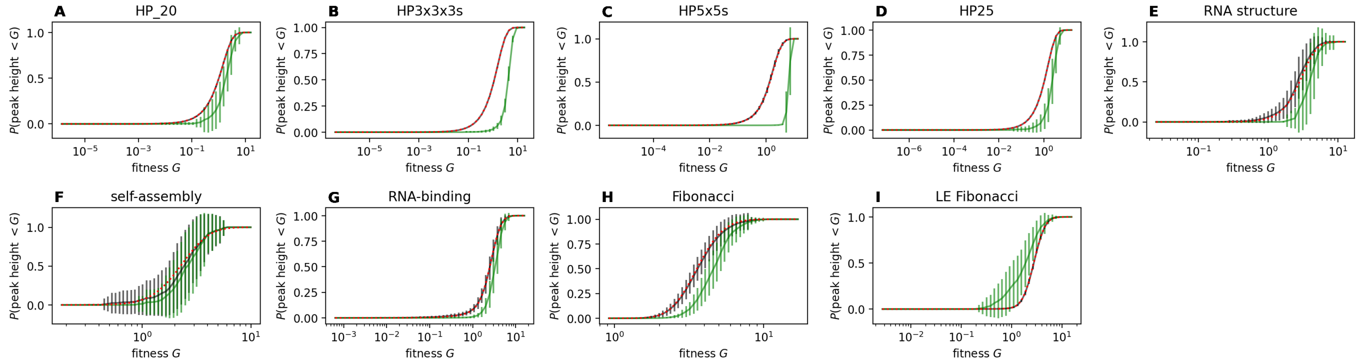

Figure S18: **Peak height distribution when the PF map is based on an exponential distribution:** same as Fig. S17, but for PF maps based on an exponential distribution with $\lambda = 1$.

In the main text, we approximated the expected CDF of peaks in a given GPF map, i.e. the fraction of

all peaks in the GPF map with fitness lower than G:

$$P_{\text{peaks}}(F \leq G) \approx \frac{1}{\sum_{\text{NC index i}}(\epsilon_i + 1)^{-1}} \sum_{\text{NC index i}} \frac{C_F(G)^{\epsilon_i+1}}{\epsilon_i + 1} \tag{17}$$

In the main text, eq. (17) was applied only to the RNA structure map, and so data for all GP maps is shown in Fig. S17 for a uniform distribution underlying the PF map ($C_F(G) = G$ for $0 \leq G \leq 1$) and Fig. S18 for an exponential distribution ($C_F(G) = 1 - \exp(-G)$). We find that eq. (17) approximates the mean CDF in simulations well. Moreover, low-fitness peaks tend to be under-represented among the peaks realised at the end of adaptive walks (but this aspect is not as clear in the self-assembly and HP_20 maps). The only clear exception is the low-evolvability version of the Fibonacci GP map, where low-fitness peaks are overrepresented among the endpoints of adaptive walks. This could be explained by the fact that the LE Fibonacci model, with its negative NC-size-evolvability relationship, is the only model, where low-fitness peaks have the largest sizes (see Figs. S13 & S14), meaning that the "arrival of the frequent" [16] effect may favour these large, low-fitness peaks. However, an alternative explanation could lie in the generally lower evolvability in this model, which means that the highest peaks may not even be accessible (as reflected in its low navigability of 0.1). Another special case is the self-assembly GP map, where the peak height CDF depends strongly on the PF map, and the difference between the two mean CDFs is particularly small compared to their variabilities. This could be an artefact of the extremely low number of 13 phenotypes in this GP map, leading to a small number of distinct fitness values in each PF map.

To analyse more carefully, which factors may be important for adaptive walks to avoid low-fitness peaks, Figs. S19& S20 show the following information: if we group peaks into bins based on their NC sizes $|NC|$ and height F, which groups are over-represented among the endpoints of adaptive walks? If evolvabilities and NC sizes were the main factor driving walks to high-fitness peaks, then this over-representation should increase with $|NC|$, even when controlling for fitness. We indeed find some evidence that high-$|NC|$ peaks may be over-represented among the endpoints of adaptive walks, compared to their low-$|NC|$ counterparts of similar fitness. However, detailed explorations of how these different factors (NC size, evolvability, fitness, as well as other NC graph features) contribute to evolutionary outcomes are a complex topic for future work. This also includes a careful analysis of zero-evolvability NCs, which always form peaks, but can never be reached.

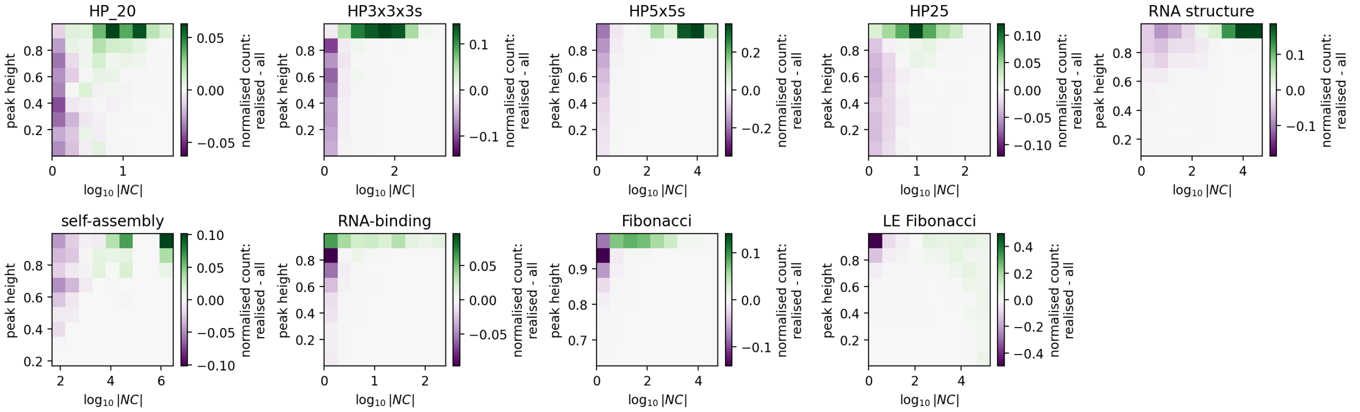

Figure S19: **Which peaks are over-represented among the endpoints of adaptive walks:** This plot aggregates data from Fig. S17: the set of all peaks and the endpoints of adaptive walks. We bin both datasets by NC size $|NC|$ (x-axis) and fitness F (y-axis). For each landscape, we compute the fraction of all peaks and the fraction of endpoints falling into each bin. The plotted histograms report the difference between these two fractions, averaged over PF maps. Thus, this plot shows in which $|NC|$-F-bin, peaks are over-represented among evolutionary endpoints (green) or under-represented (purple).

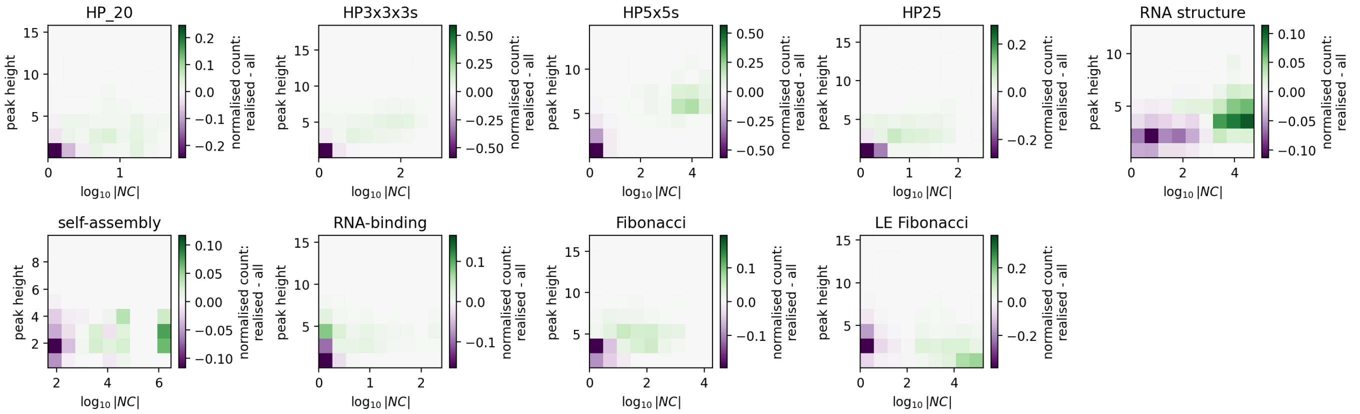

Figure S20: **Which peaks are over-represented among the endpoints of adaptive walks:** same as Fig. S19, but for the data from Fig. S18, where PF maps are based on an exponential distribution.

S2.5 Additional navigability analyses

S2.5.1 Navigable vs. non-navigable synthetic NC graphs

In this section, we use synthetic networks to test our navigability scaling from eq. (3) on NC graphs with a variety of evolvability distributions, including broader distributions than assumed in the derivation. We will do this with the simplification of NC graphs with one NC per phenotype, where network degree is the same as evolvability.

To create a test set of NC graphs, we built synthetic networks with degrees drawn from three different

types of distributions: a single degree value (i.e. a delta-function-like distribution), a Poisson distribution and a geometric distribution. For each type of distribution, we varied the number of draws and the mean, to design NC graphs with different numbers of nodes (*numbers of phenotypes* in this context) and mean degree (*mean evolvability* in this context). Specifically, we used the parameters (50, 100, 200, 300, 500, 10^3) for the number of phenotypes and ($0.005n_p$, $0.015n_p$...$0.245n_p$) for the mean degree. Once we had created a suitable list of node degrees, and after checking that the degree sum is even to ensure that a solution exists, we fed this into the configuration_model() function of the NetworkX Python package [32] to create a network with the desired degree distribution. While the resulting network satisfies the specified degree list, it may contain multi-edges and self-loops, which we removed. Finally, we discarded any isolated nodes to avoid artefacts.

Fig S21 gives an overview of the properties of the resulting networks and their navigabilities, with each row corresponding to one type of evolvability distribution. First, we analysed the evolvability distributions of the constructed networks (columns I & II in Fig S21, with one row for each type of distribution). Then, we investigated their navigabilities (column III in Fig S21). We find that low-evolvability NC graphs are non-navigable, as expected from eq. (3) (black line, for $\delta = 0.1$). Qualitatively, this holds even for the NC graphs with geometric degree distribution, even though the assumption of a single-peaked degree distribution around a well-defined mean ϵ_{NC} is no longer accurate, but we find three quantitative outliers, which are expected to have navigabilities below 0.1 based on the navigability scaling, but actually have navigabilities of up to 0.23.

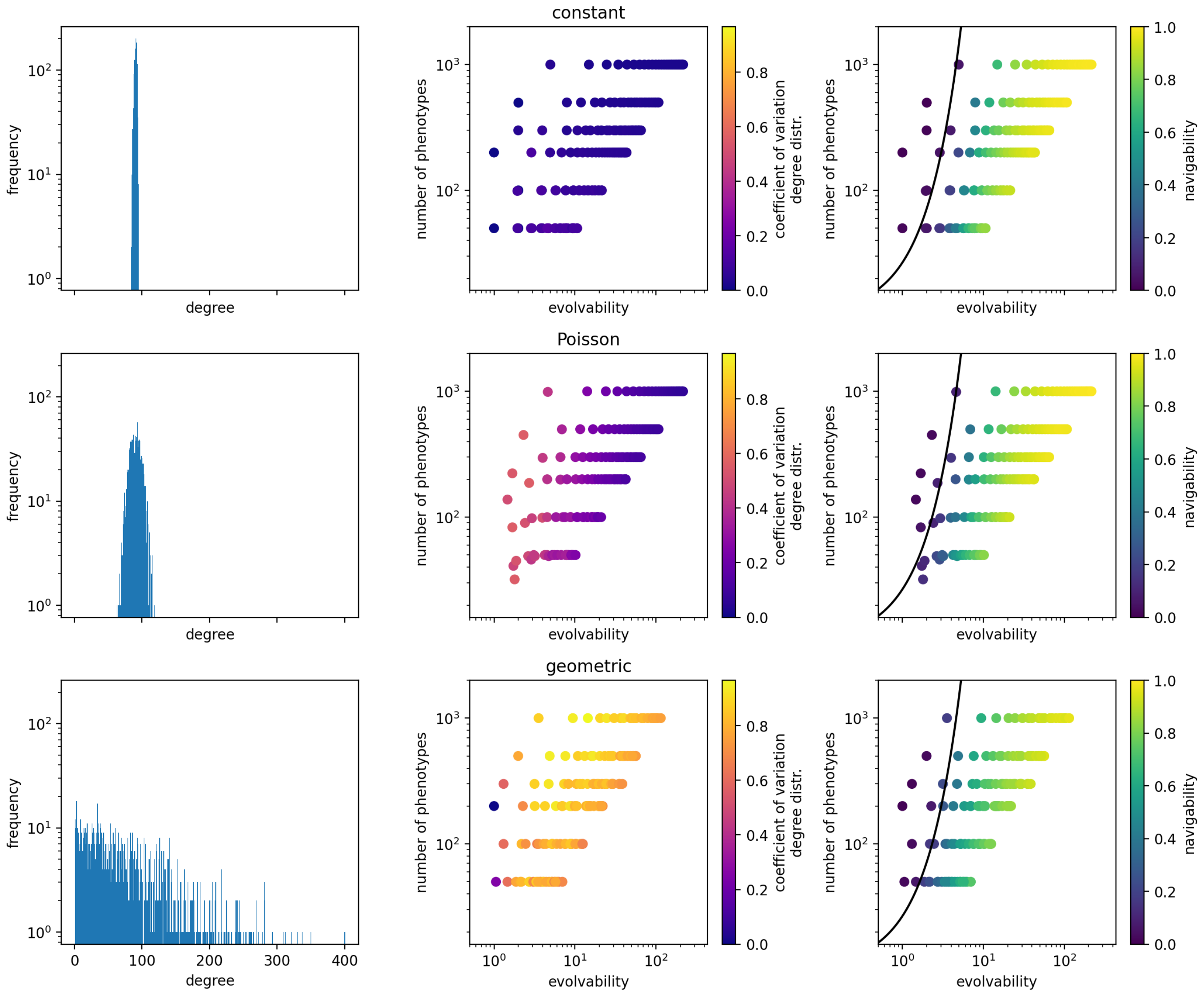

Figure S21: **Test of navigability scaling on synthetic networks:** Here we use a test set of synthetic NC graphs with degrees sampled from three different distributions: a single degree value (first row), a Poisson distribution (second row) and a geometric distribution (third row), with a variety of mean degree and number of nodes in each case. Columns I & II characterise these synthetic networks: column I shows the degree distribution for one example NC graph per distribution type. Column II plots the number of phenotypes (nodes) in each NC graph against its evolvability (geometric mean of the degree distribution), illustrating that a diverse range of networks was created for each distribution. The colour of the scatter point is given by the coefficient of variation of the NC graph's degree distribution, illustrating the difference between the different types of distributions. Finally, in the third column, the navigability of each network is indicated by the colour (based on 500 PF maps and 10 source-target pairs each). We find that low-evolvability NC graphs are non-navigable, as expected from eq. (3) (black line, for $\delta = 0.1$).

S2.5.2 Navigable vs. non-navigable GP maps in a sample of GP maps based on the Fibonacci model

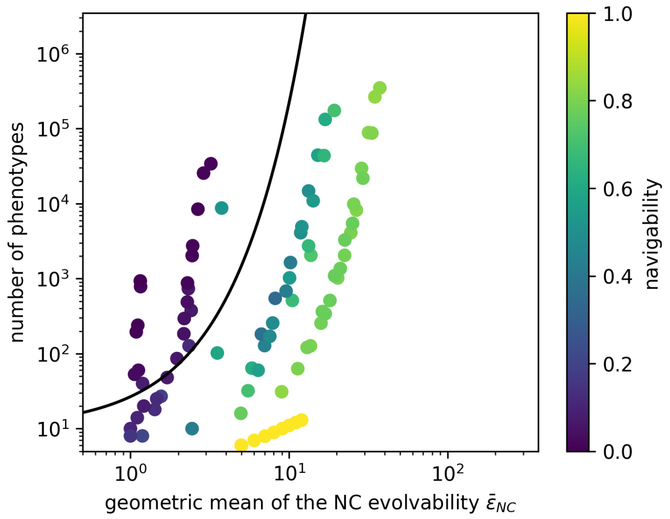

Figure S22: **Navigable vs. non-navigable GP maps in a sample of GP maps based on the Fibonacci model:** For each Fibonacci-based GP map (see text), we plot the number of phenotypes against the geometric mean of its NC evolvabilities, and indicate its navigability by the colour scale (based on 500 realisations of the PF map with 10 source-target pairs each). We find that low-evolvability GP maps are mostly non-navigable, as expected from eq. (3) (black line, $\delta = 0.1$), despite the approximations made in the derivation.

As a second test of how well our scaling from eq. (3) identifies non-navigable GP maps, we apply it to an additional test set of GP maps. Ideally, these GP maps fulfil our assumption of having a single NC per phenotype but differ in their numbers of phenotypes and evolvabilities. Thus, we work with variations of the Fibonacci model, which is simple and has a single NC per phenotype. To generate GP maps with different numbers of phenotypes, we vary their sequence length and alphabet size ($5 \leq L \leq 13$ and $2 \leq K \leq 5$, except maps with L/K combinations of 13/4, 11/5, 12/5, 13/5, which were computationally unfeasible). To create even more maps, we replaced increasing fractions of each GP map with deleterious genotypes: We created four derivative maps from each GP map, keeping a set fraction of all phenotypes in each case (10^{-3}, 10^{-2}, 10^{-1} and 0.5) and replacing the genotypes corresponding to the remaining phenotypes with deleterious genotypes. Thus, the removed phenotypes disappear from the NC graph and all derived metrics (number of phenotypes n_p and the NC evolvability distribution). If any NC has zero evolvability $\epsilon_{NC} = 0$ after this procedure, we also delete this phenotype since zero-evolvability NCs could affect navigability in non-trivial ways. We only analyse GP maps with more than five nodes in their NC graph to avoid small-size effects.

Thus, we created GP maps with different evolvabilities and number of phenotypes and computed their navigabilities through simulations. Fig S22 shows that this test set includes both navigable and non-navigable GP maps with a range of evolvabilities and numbers of phenotypes. Among these GP maps, our approximate scaling from eq. (3) successfully identifies a minimum evolvability, below which most GP maps are non-navigable ($\delta = 0.1$), with only two exceptions: one with navigability 0.12, close to our expected < 0.1, and

one special high-navigability NC graph with navigability of 0.52, where one phenotype connects to all further phenotypes, giving a star-graph with low evolvability but always one potential two-step path.

S2.5.3 Disjoint components and navigability in NC graphs

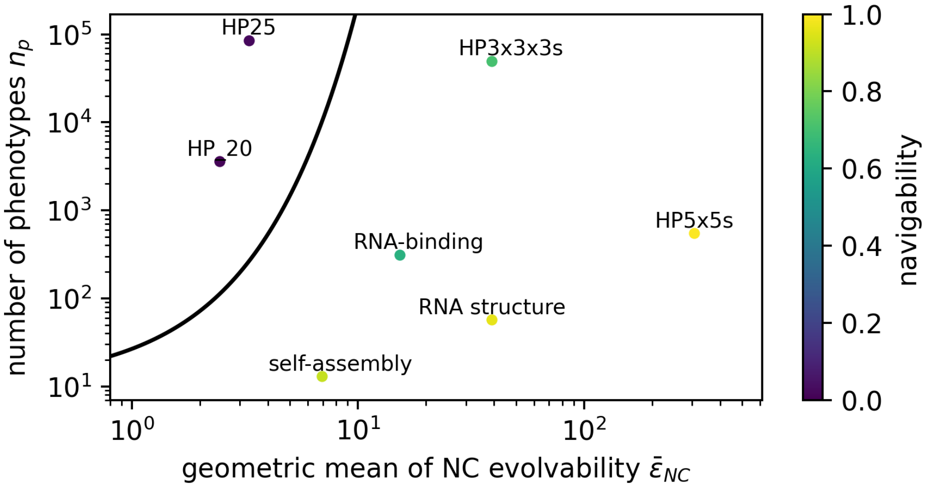

Figure S23: **Evolvability plays a key role for navigability, even when working only with the largest connected component of a NC graph:** First, every NC graph is reduced to its largest connected component, to exclude source/target pairs, where no mutational path exists (neither accessible nor non-accessible paths). Navigability is computed on this connected NC graph. As in Fig. 6 the main text, all NCs belonging to the same phenotype are first connected, before computing NC evolvabilities. Then, the number of phenotypes is plotted against the geometric mean of the evolvabilities, with navigability shown as a colour. As before, eq. (3) (black line, $\delta = 0.1$) successfully identifies an evolvability below which GP maps are non-navigable, despite relying on strong approximations.

Finally, we will investigate one potential confounding factor in the navigability analysis in the main text: the presence of disconnected, zero-evolvability NCs, which exist in several GP maps (see table 1), and cannot have accessible paths to any target phenotypes. To exclude these NCs as possible confounding factors, we reduce each NC graph to its largest connected component (largest in terms of the highest number of NCs). In this setting, any NC has a mutational path to any other NC, but this path does not have to be an accessible, fitness-increasing path (see, for example [35] for low-navigability fitness landscapes even without fragmentation into disconnected components) .

Even after reducing each NC graph to a single connected component, the navigabilities and evolvabilities of our GPF maps differ drastically (Fig. S23): in two of the HP protein models (HP_20 and HP25), evolvabilities remain low, indicating that a typical NC is only connected to a small number of different phenotypes. As expected from our approximate scaling (eq. (3)), these low-evolvability GPF maps are of low navigability even when they do not have mutationally disjoint components. The remaining GPF maps have higher evolvabilities and higher navigabilities, as before.

References

[1]S. F. Greenbury, A. A. Louis, and S. E. Ahnert, "The structure of genotype-phenotype maps makes fitness landscapes navigable", Nat. Ecol. Evol. **6**, 1742–1752 (2022).

[2]B. Schmiegelt and J. Krug, "Evolutionary accessibility of modular fitness landscapes", Journal of Statistical Physics **154**, 334–355 (2014).

[3]S. Nowak and J. Krug, "Accessibility percolation on n-trees", Europhysics Letters **101**, 66004 (2013).

[4]P. Schuster, W. Fontana, P. F. Stadler, and I. L. Hofacker, "From sequences to shapes and back: a case study in RNA secondary structures", Proceedings of the Royal Society of London. Series B: Biological Sciences **255**, 279–284 (1994).

[5]S. F. Greenbury, "General properties of genotype-phenotype maps for biological self-assembly", PhD thesis (University of Cambridge, 2014).

[6]M. Weiß and S. E. Ahnert, "Phenotypes can be robust and evolvable if mutations have non-local effects on sequence constraints", J. R. Soc. Interface **15**, 20170618 (2018).

[7]J. A. G. M. de Visser and J. Krug, "Empirical fitness landscapes and the predictability of evolution", Nat. Rev. Genet. **15**, 480–490 (2014).

[8]S. Kauffman and S. Levin, "Towards a general theory of adaptive walks on rugged landscapes", J. Theor. Biol. **128**, 11–45 (1987).

[9]S. F. Greenbury, S. Schaper, S. E. Ahnert, and A. A. Louis, "Genetic correlations greatly increase mutational robustness and can both reduce and enhance evolvability", PLOS Comput. Biol. **12**, e1004773 (2016).

[10]S. E. Ahnert, "Structural properties of genotype–phenotype maps", J. R. Soc. Interface **14**, 20170275 (2017).

[11]S. Manrubia, J. A. Cuesta, and *et. al*, "From genotypes to organisms: state-of-the-art and perspectives of a cornerstone in evolutionary dynamics", Physics of Life Reviews **38**, 55–106 (2021).

[12]I. G. Johnston, K. Dingle, S. F. Greenbury, C. Q. Camargo, J. P. Doye, S. E. Ahnert, and A. A. Louis, "Symmetry and simplicity spontaneously emerge from the algorithmic nature of evolution", PNAS **119**, e2113883119 (2022).

[13]S. F. Greenbury and S. E. Ahnert, "The organization of biological sequences into constrained and unconstrained parts determines fundamental properties of genotype-phenotype maps", Journal of The Royal Society Interface **12**, 20150724 (2015).

[14]S. Manrubia and J. A. Cuesta, "Distribution of genotype network sizes in sequence-to-structure genotype - phenotype maps", Journal of The Royal Society Interface **14**, 20160976 (2017).

[15]K. Dingle, S. Schaper, and A. A. Louis, "The structure of the genotype–phenotype map strongly constrains the evolution of non-coding RNA", Interface focus **5**, 20150053 (2015).

[16]S. Schaper and A. A. Louis, "The arrival of the frequent: how bias in genotype-phenotype maps can steer populations to local optima", PloS one **9**, e86635 (2014).

[17]S. Schaper, "On the significance of neutral spaces in adaptive evolution", PhD Thesis (Oxford University, UK, 2012).

[18]C. A. Macken and A. S. Perelson, "Protein evolution on rugged landscapes.", PNAS **86**, 6191–6195 (1989).

[19]S. Schaper, I. G. Johnston, and A. A. Louis, "Epistasis can lead to fragmented neutral spaces and contingency in evolution", Proceedings of the Royal Society B: Biological Sciences **279**, 1777–1783 (2012).

[20]M. Weiß and S. E. Ahnert, "Using small samples to estimate neutral component size and robustness in the genotype–phenotype map of RNA secondary structure", Journal of the Royal Society Interface **17**, 20190784 (2020).

[21]M. Weiß, "Structural properties of genotype-phenotype maps: models, estimates and evolution", PhD thesis (Apollo - University of Cambridge Repository, 2020).

[22]H. Li, R. Helling, C. Tang, and N. Wingreen, "Emergence of preferred structures in a simple model of protein folding", Science **273**, 666–669 (1996).

[23]A. Irbäck and C. Troein, "Enumerating designing sequences in the HP model", Journal of Biological Physics **28**, 1–15 (2002).

[24]S. F. Greenbury, I. G. Johnston, A. A. Louis, and S. E. Ahnert, "A tractable genotype–phenotype map modelling the self-assembly of protein quaternary structure", J. R. Soc. Interface **11**, 20140249 (2014).

[25]D. Doty, "Theory of algorithmic self-assembly", Communications of the ACM **55**, 78–88 (2012).

[26]R. Lorenz, S. H. Bernhart, C. Höner zu Siederdissen, H. Tafer, C. Flamm, P. F. Stadler, and I. L. Hofacker, "ViennaRNA package 2.0", Algorithms for molecular biology **6**, 1–14 (2011).

[27]J. L. Payne, F. Khalid, and A. Wagner, "RNA-mediated gene regulation is less evolvable than transcriptional regulation", PNAS **115**, E3481–E3490 (2018).

[28]S. Greenbury and S. E. Ahnert, "The organization of biological sequences into constrained and unconstrained parts determines fundamental properties of genotype–phenotype maps", J. R. Soc. Interface **12**, 20150724 (2015).

[29]A. Wagner, "Robustness and evolvability: a paradox resolved", Proceedings of the Royal Society B: Biological Sciences **275**, 91–100 (2008).

[30]E. Ferrada and A. Wagner, "A comparison of genotype-phenotype maps for RNA and proteins", Biophysical journal **102**, 1916–1925 (2012).

[31]R. A. Goldstein, "The structure of protein evolution and the evolution of protein structure", Current opinion in structural biology **18**, 170–177 (2008).

[32]A. Hagberg, P. J. Swart, and D. A. Schult, *Exploring network structure, dynamics, and function using networkx*, tech. rep. (Los Alamos National Laboratory (LANL), Los Alamos, NM (United States), 2008).

[33]M. Newman, *Networks* (Oxford University Press, 2018).

[34]J. Aguirre, J. M. Buldú, M. Stich, and S. C. Manrubia, "Topological structure of the space of phenotypes: the case of RNA neutral networks", PloS one **6**, e26324 (2011).

[35]J. Franke, A. Klozer, J. de Visser, and J. Krug, "Evolutionary accessibility of mutational pathways", PLOS Comput. Biol. **7** (2011).
